# The effects of cinnamon on patients with metabolic diseases: an umbrella review of meta-analyses of randomized controlled trials

**DOI:** 10.3389/fnut.2025.1683477

**Published:** 2025-11-03

**Authors:** Haobo Gou, Ling Zhong, Qiuya Wei, Yong Fan

**Affiliations:** ^1^Department of General Surgery, Lanzhou University Second Hospital, Lanzhou University Second Clinical Medical College, Lanzhou, China; ^2^Department of Dermatology, Sichuan Provincial People's Hospital, University of Electronic Science and Technology of China, Chengdu, China

**Keywords:** cinnamon, metabolic diseases, umbrella, meta-analysis, systematic review

## Abstract

**Introduction:**

Animal and clinical studies have demonstrated a range of potential health benefits associated with cinnamon. However, its effects on metabolic parameters such as blood glucose, blood pressure, lipid profiles, and body weight in patients with metabolic diseases remain controversial. To systematically assess the current evidence, we conducted an umbrella review of meta-analyses to comprehensively evaluate the impact of cinnamon supplementation on metabolic outcomes in patients with metabolic diseases.

**Methods:**

A systematic search was performed in PubMed, Embase, Web of Science, Scopus, and the Cochrane Library to identify relevant systematic reviews and meta-analyses of randomized placebo-controlled trials investigating cinnamon supplementation in individuals with metabolic diseases. The methodological quality and strength of evidence were assessed using AMSTAR 2 tool (A MeaSurement Tool to Assess systematic Reviews, version 2).

**Results:**

A total of 21 meta-analyses comprising 139 comparisons, were included for qualitative synthesis. The findings indicate that cinnamon supplementation is significantly associated with improvements in fasting blood glucose and lipid profiles, with more pronounced effects observed in patients with diabetes and metabolic syndrome. Subgroup analyses suggest that higher doses (>1.5 g/day) and shorter intervention durations (≤2 months) may enhance these benefits. Additionally, cinnamon shows potential in modulating insulin resistance, antioxidant capacity, and blood pressure regulation.

**Conclusion:**

These results underscore the promising role of cinnamon as an adjunctive therapy for metabolic diseases. Future research should focus on well-designed randomized controlled trials with extended follow-up periods to further confirm its efficacy and elucidate underlying mechanisms, thereby providing robust evidence for clinical and public health applications.

**Systematic review registration:**

https://www.crd.york.ac.uk/PROSPERO/view/CRD420251010073, identifier: CRD420251010073.

## 1 Introduction

Metabolic diseases comprise a group of conditions characterized by disturbances in glucose, lipid, or protein metabolism (1). Common examples include type 2 diabetes, hypertension, obesity, hyperlipidemia, hyperuricemia/gout, non-alcoholic fatty liver disease, and metabolic syndrome (1). In recent years, the prevalence of metabolic diseases has been rising continuously and has reached alarming levels, posing a significant global public health burden (2). Currently, over 890 million adults worldwide are diagnosed with obesity, and more than 589 million are living with diabetes (3, 4).

*Cinnamomum*, a genus in the Lauraceae family, is widely used not only as a culinary spice but also in traditional herbal medicine (5, 6). The spice cinnamon is obtained from plants of the genus *Cinnamomum*. Preclinical and clinical studies have demonstrated that cinnamon possesses diverse pharmacological properties, including antioxidant, anti-inflammatory, antitumor, immunomodulatory, antidiabetic, and lipid-lowering effects (7, 8).

Although numerous studies have reported beneficial metabolic outcomes associated with cinnamon supplementation in individuals with metabolic diseases (9–11), findings across trials remain inconsistent. Therefore, an umbrella review is warranted to systematically evaluate and synthesize evidence from existing systematic reviews and meta-analyses. This study aims to assess the overall effects of cinnamon on metabolic outcomes in patients with metabolic diseases. Additionally, we seek to explore whether the effectiveness of cinnamon varies according to dosage, duration of intervention, or underlying disease type, thereby providing more robust evidence to guide clinical practice and future research.

## 2 Materials and methods

This umbrella review was prospectively registered with PROSPERO (CRD420251010073). The study adhered to the Preferred Reporting Items for Systematic Reviews and Meta-Analyses (PRISMA) guidelines (12).

### 2.1 Literature search strategy

A comprehensive search was conducted in PubMed, Web of Science, Embase, Scopus, and the Cochrane Library up to March 2025 to identify systematic reviews and meta-analyses investigating the effects of cinnamon supplementation on metabolic diseases. The search terms included: (“*Cinnamomum zeylanicum*” OR “*Cinnamomum verum*” OR “Cinnamon” OR “Cinnamons”) AND (“Systematic Review” OR “Meta-Analysis” OR “systematic literature review” OR “meta-analysis”). During study selection, only studies conducted in patients with metabolic diseases—including diabetes, metabolic syndrome, polycystic ovary syndrome (PCOS), Non-Alcoholic Fatty Liver Disease (NAFLD), hypertension and related diseases—were included. No language restrictions were applied. Relevant studies were identified through screening of titles, abstracts, and full texts. Non-English articles meeting the inclusion criteria were included, with data extraction assisted by translation tools (e.g., DeepL, ChatGPT) when necessary.

### 2.2 Eligibility and inclusion/exclusion criteria

Inclusion criteria were as follows:

(1) Adults aged ≥18 years with diagnosed metabolic diseases (e.g., diabetes, PCOS, NAFLD, metabolic syndrome, hypertension), (2) The intervention involved supplementation with cinnamon or cinnamon extract, with cinnamon used as the sole intervention, either as a dietary supplement or a culinary spice, (3) Placebo-controlled comparisons, (4) At least one outcome reported among: fasting blood glucose (FBG), glycated hemoglobin (HbA1c), Homeostatic Model Assessment for Insulin Resistance (HOMA-IR), total cholesterol (CHOL), triglycerides (TG), high-density lipoprotein (HDL), low-density lipoprotein (LDL), systolic blood pressure (SBP), diastolic blood pressure (DBP), body weight (BW), or body mass index (BMI), (5) Meta-analyses reporting effect sizes (MD, WMD, or SMD) with 95% confidence intervals (CIs).

Exclusion criteria: (1) Systematic reviews without quantitative synthesis, (2) Meta-analyses lacking effect sizes with 95% CIs or with incomplete data, (3) Studies using cinnamon combined with other supplements, (4) Meta-analyses based solely on observational studies.

### 2.3 Data extraction, quality assessment, and publication bias

We used EndNote software to remove duplicate records during the study selection process. Two reviewers independently extracted data, with a third reviewer verifying accuracy. Disagreements were resolved by consultation with a fourth researcher. Duplicate records were removed and references were managed using EndNote software during the study selection process. Extracted data included: first author, year of publication, number of included studies, study design, population characteristics, intervention details, outcome measures, total sample size, effect estimates with 95% CIs, heterogeneity statistics (*I*^2^), and statistical models used. For primary studies within each meta-analysis, we extracted author, sample size, outcome type, group sample sizes, and pre/post means with SDs for further re-analysis.

Methodological quality was assessed using the AMSTAR-2 tool. Publication bias was evaluated with Egger's test, and studies with significant bias were adjusted using the Trim and Fill method. Sensitivity analyses were performed to assess the robustness of the findings (13).

### 2.4 Statistical analysis

Statistical analyses were performed using Stata (version 15.0) and R Studio (version 4.3.2). Except where specified, two-tailed *p*-values < 0.05 were considered statistically significant.

For all included studies, we extracted mean changes and SDs for outcomes before and after intervention in both intervention and control groups to estimate overall mean differences as effect sizes. When not directly reported, mean changes were calculated as post-intervention minus baseline values, SDs were computed as √[(baseline SD^2^ + endpoint SD^2^) – 2R × baseline SD × endpoint SD] assuming a correlation coefficient (*R*) of 0.5. If only SE was reported (14), SD was calculated as SE × √*n* (*n* = sample size per group). For studies reporting medians with ranges or 95% CIs, means and SDs were estimated using standard formulas. All meta-analyses were synthesized using standardized mean differences (SMDs).

For each eligible meta-analysis, both fixed- and random-effects models were applied to calculate pooled SMDs with 95% CIs (15). Studies with incomplete data were excluded to ensure accuracy. Between-study heterogeneity was assessed via *I*^2^ and its 95% CI (16, 17). Prediction intervals (PIs) under random-effects models were also computed to assess the likely range of true effects in future studies (18).

Publication bias was assessed using Egger's test, with *p* < 0.05 suggesting small-study effects (19). To explore sources of heterogeneity, subgroup analyses were conducted based on cinnamon dose, intervention duration, and disease type. Adverse events related to cinnamon supplementation were also summarized.

Excess significance bias (20, 21) was evaluated by comparing the observed number of significant studies (O, *p* < 0.05) with the expected number (E). The E-value was calculated as the sum of statistical power across all included studies, with power estimated based on the effect size from the largest study in each meta-analysis using a non-central t-distribution. A *p*-value < 0.10 was considered indicative of excess significance bias.

### 2.5 Assessment of evidence credibility

The strength of evidence was classified according to the following criteria (22–24): (1) *p* < 10^−6^ in random-effects meta-analysis, (2) total sample size >1,000, (3) *p* < 0.05 in the largest individual study, (4) *I*^2^ < 50%, (5) no evidence of small-study effects, (6) 95% PI excluding the null value, (7) no excess significance bias. Associations meeting all seven criteria were considered convincing. Evidence was deemed highly suggestive if criteria (1–3) were met, suggestive if only *p* ≤ 0.001 and sample size >1,000 were satisfied, weak if only *p* ≤ 0.05 was met, and non-significant if *p* > 0.05. The results of the Evidence Credibility assessment are presented in **Tables 2** and **3** and **Figures 3**–**13**.

### 2.6 Overlap assessment and strategy for handling overlapping meta-analyses

Corrected Covered Area (CCA) was used to quantify overlap between included meta-analyses (25):


$$\begin{array}{l} \text{CCA}=\frac{N_{\text{r}}\;-\;N_{\text{s}}}{\left(R^{*}N_{s}\;-\;\right)N_{s}} \end{array}$$

where Nr is the total number of primary study occurrences (including duplicates), Ns is the number of unique studies, and *R* is the number of meta-analyses. Overlap was categorized as slight (0%−5%), moderate (6%−10%), high (11%−15%), or very high (>15%) (25). This helps identify redundancy and risk of bias from duplicate evidence.

For high overlap (CCA ≥ 6%), two strategies were used (26–28): (1) selecting the most recent, comprehensive, or methodologically robust meta-analysis (via AMSTAR-2), (2) extracting all relevant primary studies for a *de novo* meta-analysis. When overlap was slight (CCA ≤ 5%), existing pooled estimates were directly used (29).

## 3 Results

**Figure 1:**
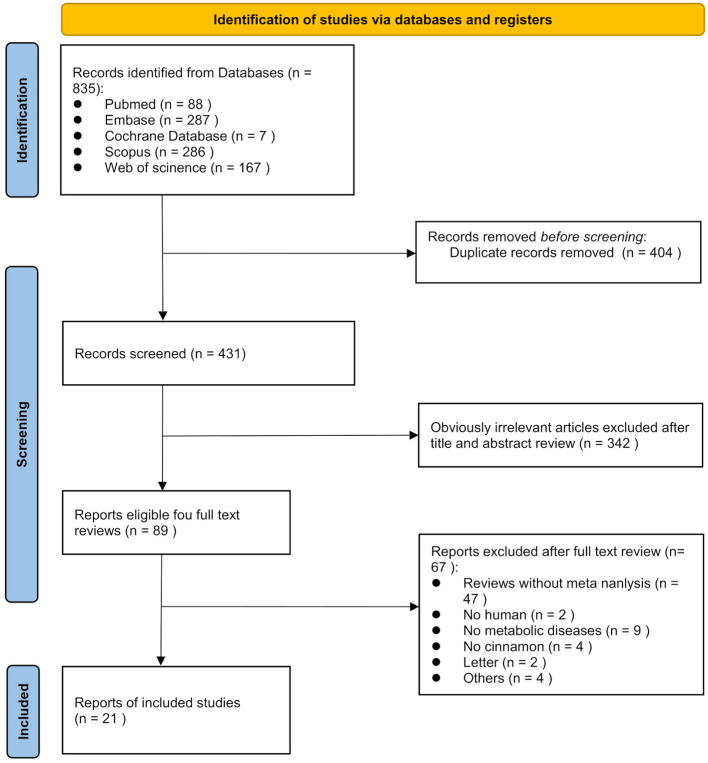
Flow chart of the literature search.

**Figure 2:**
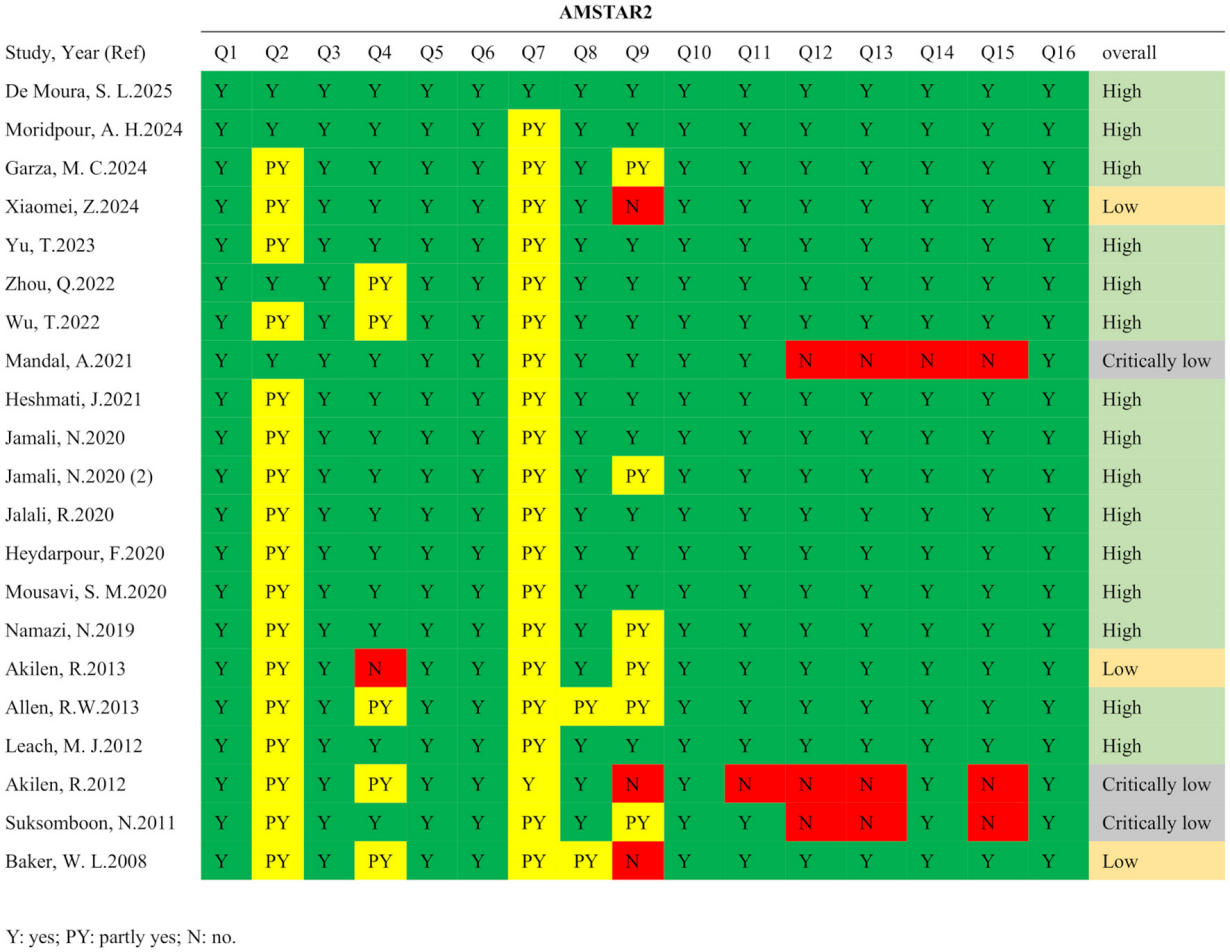
Results of risk of bias assessment based on AMSTAR 2 tool.

According to the PRISMA guidelines, the literature screening process of this study is presented in Figure 1. A total of 835 records were initially identified through systematic searches of the selected electronic databases. After screening the titles, abstracts, and full texts, and excluding studies that did not meet the inclusion criteria, 21 meta-analyses comprising 139 comparisons were ultimately included. The detailed characteristics of the included studies are summarized in Table 1. The standardized mean differences (SMDs) under the random-effects model, corresponding *p*-values, and heterogeneity measures from the included meta-analyses are presented in Table 2. These publications were dated from 2008 to 2025. The cinnamon supplementation dose in the included studies ranged from 0.12 to 6 grams per day, with intervention durations varying from 1.5 to 12 months. Regarding the methodological quality assessment, the AMSTAR 2 tool was used to evaluate all included studies. Among the 21 meta-analyses, 15 were rated as high quality, three as low quality, and three as critically low quality (Figure 2).

**Table 1:** Summary of the characteristics of the included meta-analyses.

**Study, year (Ref)**	**Number of study^a^**	**Study design**	**Exposure**	**Dosage**	**Duration**	**People**	**Outcomes**	**Total^a^**	**Type of metrics**	**Summary effect size (95% CI)**
Mandal, 2021a (76)	7	RCT	Cinnamon			Type 2 diabetes	FBG	389	MD	−12.60 (−27.57, 2.37)
Mandal, 2021b (76)	7	RCT	Cinnamon			Type 2 diabetes	HbA1c	478	MD	0.01 (−0.11, 0.13)
Mandal, 2021c (76)	6	RCT	Cinnamon			Type 2 diabetes	TG	319	MD	−20.47 (−46.07, 5.14)
Mandal, 2021d (76)	6	RCT	Cinnamon			Type 2 diabetes	CHOL	319	MD	−3.91 (−14.37, 6.55)
Mandal, 2021e (76)	6	RCT	Cinnamon			Type 2 diabetes	LDL	319	MD	0.24 (2.22, 2.70)
Mandal, 2021f (76)	4	RCT	Cinnamon			Type 2 diabetes	HDL	242	MD	1.03 (−1.91, 3.97)
Baker, 2008a (77)	4	RCT	Cinnamon			Diabetes	HbA1c	204	WMD	0.07 (−0.11, 0.26)
Baker, 2008b (77)	4	RCT	Cinnamon			Diabetes	FBG	207	WMD	−17.15 (−47.58, 13.27)
Baker, 2008c (77)	4	RCT	Cinnamon			Diabetes	CHOL	207	WMD	−9.63 (−35.94, 16.67)
Baker, 2008d (77)	4	RCT	Cinnamon			Diabetes	TG	207	WMD	−28.44 (−61.81, 4.94)
Baker, 2008e (77)	3	RCT	Cinnamon			Diabetes	HDL	147	WMD	1.58 (−0.74, 3.89)
Baker, 2008f (77)	4	RCT	Cinnamon			Diabetes	LDL	207	WMD	−4.71 (−18.12, 8.71)
Baker, 2008g (77)	3	RCT	Cinnamon			Type 2 diabetes	HbA1c	147	WMD	0.01 (−0.20, 0.22)
Leach, 2012a (65)	8	RCT	Cinnamon			Diabetes	FBG	338	MD	−0.83 (−1.67, 0.02)
Leach, 2012b (65)	4	RCT	Cinnamon			Diabetes	adverse events	264	RR	0.83 (0.22, 3.07)
Leach, 2012c (65)	6	RCT	Cinnamon		≥12 weeks	Diabetes	HbA1c	405	MD	−0.06 (−0.29, 0.18)
Leach, 2012e (65)	3	RCT	Cinnamon	≤ 1 g/day		Diabetes	FBG	116	MD	−1.35 (−3.71, 1.01)
Leach, 2012f (65)	4	RCT	Cinnamon	1.5–2 g/day		Diabetes	FBG	157	MD	−0.51 (−1.57, 0.56)
Leach, 2012g (65)	2	RCT	Cinnamon	3 g/day		Diabetes	FBG	85	MD	−1.72 (−4.80, 1.36)
Leach, 2012h (65)	4	RCT	Cinnamon		< 12 weeks	Diabetes	FBG	99	MD	−1.74 (−3.89, 0.41)
Leach, 2012i (65)	4	RCT	Cinnamon		≥12 weeks	Diabetes	FBG	239	MD	−0.13 (−0.64, 0.38)
Leach, 2012j (65)	3	RCT	Cinnamon	1 g/day		Diabetes	HbA1c	222	MD	−0.1 (−0.51, 0.31)
Leach, 2012k (65)	5	RCT	Cinnamon			Type 2 diabetes	HbA1c	348	MD	−0.10 (−0.38, 0.18)
Zhou, 2022a (78)	15	RCT	Cinnamon			Diabetes	FBG	890	SMD	−0.54 (−0.68, −0.40)
Zhou, 2022b (78)	7	RCT	Cinnamon			Diabetes	BMI	488	SMD	−0.73 (−0.94, −0.51)
Zhou, 2022d (78)	16	RCT	Cinnamon			Diabetes	HbA1c	1,354	SMD	−0.63 (−0.77, −0.49)
Zhou, 2022e (78)	4	RCT	Cinnamon			Diabetes	HOMA-IR	375	SMD	−0.80 (1.06, −0.54)
Zhou, 2022f (78)	11	RCT	Cinnamon			Diabetes	CHOL	676	SMD	−0.25 (−0.42, −0.07)
Zhou, 2022g (78)	11	RCT	Cinnamon			Diabetes	LDL	664	SMD	−0.55 (−0.72, −0.39)
Zhou, 2022h (78)	11	RCT	Cinnamon			Diabetes	HDL	664	SMD	0.57 (0.41, 0.74)
Zhou, 2022i (78)	11	RCT	Cinnamon			Diabetes	TG	664	SMD	−0.6 (−0.76, −0.44)
Wu, 2022a (9)	13	RCT	Cinnamon			Metabolic syndrome	CHOL	793	WMD	−0.19 (−0.24, −0.14)
Wu, 2022b (9)	13	RCT	Cinnamon			Metabolic syndrome	TG	793	WMD	−0.10 (−0.16, −0.04)
Wu, 2022c (9)	13	RCT	Cinnamon			Metabolic syndrome	HDL	793	WMD	−0.01 (−0.02, 0.00)
Wu, 2022d (9)	13	RCT	Cinnamon			Metabolic syndrome	LDL	793	WMD	−0.16 (−0.20, −0.11)
Jamali, 2020a (79)	19	RCT	Cinnamon			Type 2 diabetes	TG	1,025	WMD	−26.27 (−38.93, −13.61)
Jamali, 2020b (79)	19	RCT	Cinnamon			Type 2 diabetes	CHOL	1,025	WMD	−13.93 (−25.64, −2.22)
Jamali, 2020c (79)	18	RCT	Cinnamon			Type 2 diabetes	LDL	958	WMD	−6.13 (−10.73, −1.54)
Jamali, 2020d (79)	16	RCT	Cinnamon			Type 2 diabetes	HDL	958	WMD	0.64 (−0.18, 1.47)
Jamali, 2020a (2, 10)	5	RCT	Cinnamon			Type 2 diabetes	SBP	332	SMD	−0.53 (−1.03, −0.03)
Jamali, 2020b (2, 10)	5	RCT	Cinnamon			Type 2 diabetes	DBP	332	SMD	0.68 (−1.30, −0.07)
Jamali, 2020c (2, 10)	7	RCT	Cinnamon			Type 2 diabetes	BW	491	SMD	−0.31 (−0.79, 0.17)
Jamali, 2020d (2, 10)	7	RCT	Cinnamon			Type 2 diabetes	BMI	491	SMD	−0.55 (−1.24, 0.14)
Jamali, 2020f (2, 10)	2	RCT	Cinnamon		3 months	Type 2 diabetes	SBP	117	SMD	−1.06 (−2.20, 0.08)
Jamali, 2020g (2, 10)	3	RCT	Cinnamon		2 months	Type 2 diabetes	SBP	215	SMD	−0.25 (−0.52, 0.02)
Jamali, 2020h (2, 10)	2	RCT	Cinnamon		3 months	Type 2 diabetes	DBP	117	SMD	−0.40 (−0.98, 0.18)
Jamali, 2020i (2, 10)	3	RCT	Cinnamon		2 months	Type 2 diabetes	DBP	215	SMD	−0.87 (−1.89, 0.15)
Jamali, 2020j (2, 10)	4	RCT	Cinnamon		3 months	Type 2 diabetes	BW	255	SMD	−0.56 (−1.27, 0.15)
Jamali, 2020k (2, 10)	3	RCT	Cinnamon		2 months	Type 2 diabetes	BW	176	SMD	0.04 (−0.25, 0.34)
Jamali, 2020l (2, 10)	4	RCT	Cinnamon		3 months	Type 2 diabetes	BMI	315	SMD	−0.82 (−1.92, 0.27)
Jamali, 2020m (2, 10)	3	RCT	Cinnamon		2 months	Type 2 diabetes	BMI	176	SMD	−0.13 (−0.43, 0.17)
Jalali, 2020a (80)	5	RCT	Cinnamon			Type 2 diabetes	SBP	332	MD	−0.53 (−1.03, −0.02)
Jalali, 2020b (80)	5	RCT	Cinnamon			Type 2 diabetes	DBP	332	MD	−0.68 (−1.30, −0.07)
Jalali, 2020c (80)	3	RCT	Cinnamon	>1.7 g/day		Type 2 diabetes	SBP	174	MD	−0.575 (−1.587, 0.437)
Jalali, 2020d (80)	2	RCT	Cinnamon	< 1.7 g/day		Type 2 diabetes	SBP	158	MD	−0.779 (−0.779, 0.147)
Jalali, 2020e (80)	3	RCT	Cinnamon	>1.7 g/day		Type 2 diabetes	DBP	174	MD	−0.976 (−1.94, −0.012)
Jalali, 2020f (80)	2	RCT	Cinnamon	< 1.7 g/day		Type 2 diabetes	DBP	158	MD	−0.292 (−0.606, 0.022)
Akilen, 2013a (81)	3	RCT	Cinnamon			Type 2 and prediabetes	SBP	139	WMD	−5.39 (−6.89, −3.89)
Akilen, 2013b (81)	3	RCT	Cinnamon			Type 2 and prediabetes	DBP	139	WMD	−2.6 (−4.53, −0.06)
Akilen, 2013c (81)	2	RCT	Cinnamon			Type 2 diabetes	SBP	117	WMD	−5.02 (−6.55, −3.49)
Akilen, 2013d (81)	2	RCT	Cinnamon			Type 2 diabetes	DBP	117	WMD	−2.64 (−4.63, −0.64)
Allen, 2013a (11)	9	RCT	Cinnamon			Type 2 diabetes	HbA1c	519	WMD	−0.16 (−0.39, 0.06)
Allen, 2013b (11)	12	RCT	Cinnamon			Type 2 diabetes	FBG	484	WMD	−24.59 (−40.52, −8.67)
Allen, 2013c (11)	12	RCT	Cinnamon			Type 2 diabetes	CHOL	484	WMD	−15.60 (−29.76, −1.44)
Allen, 2013d (11)	11	RCT	Cinnamon			Type 2 diabetes	LDL	424	WMD	−9.42 (−17.21, −1.63)
Allen, 2013e (11)	9	RCT	Cinnamon			Type 2 diabetes	HDL	424	WMD	1.66 (1.09, 2.24)
Allen, 2013f (11)	12	RCT	Cinnamon			Type 2 diabetes	TG	484	WMD	−29.59 (−48.27, −10.91)
Yu, 2023a (82)	13	RCT	Cinnamon			Type 2 diabetes	FBG	885	WMD	−4.95 (−11.27, 1.36)
Yu, 2023b (82)	12	RCT	Cinnamon			Type 2 diabetes	HbA1c	689	WMD	−0.02 (−0.14, 0.11)
Yu, 2023c (82)	10	RCT	Cinnamon			Type 2 diabetes	TG	626	WMD	−7.31 (−12.37, −2.25)
Yu, 2023d (82)	9	RCT	Cinnamon			Type 2 diabetes	CHOL	501	WMD	0.25 (−4.17, 4.66)
Yu, 2023e (82)	11	RCT	Cinnamon			Type 2 diabetes	HDL	684	WMD	1.53 (1.01, 2.05)
Yu, 2023f (82)	8	RCT	Cinnamon			Type 2 diabetes	LDL	537	WMD	−6.82 (−11.24, −2.40)
Yu, 2023g (82)	8	RCT	Cinnamon	≥1.2 g/day		Type 2 diabetes	FBG	542	WMD	−2.09 (−10.34, 6.16)
Yu, 2023h (82)	6	RCT	Cinnamon	< 1.2 g/day		Type 2 diabetes	FBG	343	WMD	−10.05 (−18.07, −2.93)
Yu, 2023i (82)	8	RCT	Cinnamon		≥8 weeks	Type 2 diabetes	FBG	606	WMD	−2.09 (−10.34, 6.16)
Yu, 2023j (82)	6	RCT	Cinnamon		< 8 weeks	Type 2 diabetes	FBG	279	WMD	−10.05 (−18.07, −2.93)
De Moura, 2025a (83)	26	RCT	Cinnamon			Type 2 diabetes	FBG	1,757	WMD	−15.26 (−22.23, −8.30)
De Moura, 2025b (83)	15	RCT	Cinnamon	≤ 2 g/day		Type 2 diabetes	FBG	1,079	WMD	−12.70 (−21.16, −4.24)
De Moura, 2025c (83)	11	RCT	Cinnamon	>2 g/day		Type 2 diabetes	FBG	678	WMD	−20.21 (−33.87, −6.54)
De Moura, 2025d (83)	22	RCT	Cinnamon			Type 2 diabetes	HbA1c	1,244	WMD	−0.56 (−0.99, −0.13)
De Moura, 2025e (83)	13	RCT	Cinnamon	≤ 2 g/day		Type 2 diabetes	HbA1c	768	WMD	−0.68 (−1.16, −0.19)
De Moura, 2025f (83)	9	RCT	Cinnamon	>2 g/day		Type 2 diabetes	HbA1c	588	WMD	−0.39 (−1.24, 0.46)
De Moura, 2025g (83)	6	RCT	Cinnamon			Type 2 diabetes	HOMA-IR	448	WMD	−0.62 (−1.29, 0.05)
De Moura, 2025h (83)	18	RCT	Cinnamon			Type 2 diabetes	CHOL	1,130	WMD	−7.46 (−18.40, 3.49 )
De Moura, 2025i (83)	11	RCT	Cinnamon	≤ 2 g/day		Type 2 diabetes	CHOL	709	WMD	−11.55 (−25.09, 1.99)
De Moura, 2025j (83)	7	RCT	Cinnamon	>2 g/day		Type 2 diabetes	CHOL	221	WMD	−1.22 (−20.84, 18.39)
De Moura, 2025k (83)	15	RCT	Cinnamon			Type 2 diabetes	HDL	1,070	WMD	2.83 (−0.9, 6.56)
De Moura, 2025l (83)	10	RCT	Cinnamon	≤ 2 g/day		Type 2 diabetes	HDL	689	WMD	0.4 (−2.73, 3.52)
De Moura, 2025m (83)	5	RCT	Cinnamon	>2 g/day		Type 2 diabetes	HDL	181	WMD	7.16 (−3.11, 17.43)
De Moura, 2025n (83)	17	RCT	Cinnamon			Type 2 diabetes	LDL	1,058	WMD	−3.58 (−9.14, 1.98)
De Moura, 2025o (83)	10	RCT	Cinnamon	≤ 2 g/day		Type 2 diabetes	LDL	637	WMD	−2.34 (−8.77, 4.08)
De Moura, 2025p (83)	7	RCT	Cinnamon	>2 g/day		Type 2 diabetes	LDL	221	WMD	−4.75 (−16.81, 7.31)
De Moura, 2025q (83)	18	RCT	Cinnamon			Type 2 diabetes	TG	1,130	WMD	−10.29 (−25.10, 4.52)
De Moura, 2025r (83)	11	RCT	Cinnamon	≤ 2 g/day		Type 2 diabetes	TG	709	WMD	−17.56 (−35.40, 0.28)
De Moura, 2025s (83)	7	RCT	Cinnamon	>2 g/day		Type 2 diabetes	TG	221	WMD	−0.25 (−23.24, 22.75)
De Moura, 2025t (83)	14	RCT	Cinnamon			Type 2 diabetes	BMI	1,254	WMD	−0.47 (−1.03, 0.09)
De Moura, 2025u (83)	9	RCT	Cinnamon	≤ 2 g/day		Type 2 diabetes	BMI	680	WMD	−1.18 (−1.97, 4.39)
De Moura, 2025v (83)	5	RCT	Cinnamon	>2 g/day		Type 2 diabetes	BMI	395	WMD	0.24 (−0.55, 1.03)
De Moura, 2025w (83)	6	RCT	Cinnamon			Type 2 diabetes	BW	451	WMD	−1.05 (−3.51, 1.41)
De Moura, 2025x (83)	3	RCT	Cinnamon	≤ 2 g/day		Type 2 diabetes	BW	256	WMD	−0.59 (−3.7, 2.52)
De Moura, 2025y (83)	3	RCT	Cinnamon	>2 g/day		Type 2 diabetes	BW	195	WMD	−1.81 (−5.82, 2.21)
Namazi, 2019a (51)	21	RCT	Cinnamon			Type 2 diabetes	FBG	1,250	WMD	−19.26 (−28.08, −10.45)
Namazi, 2019b (51)	14	RCT	Cinnamon			Type 2 diabetes	HbA1c	884	WMD	−0.24 (−0.48, −0.01)
Namazi, 2019c (51)	4	RCT	Cinnamon			Type 2 diabetes	BW	234	WMD	0.46 (−1.87, 2.80)
Namazi, 2019d (51)	5	RCT	Cinnamon			Type 2 diabetes	BMI	294	WMD	−0.05 (−0.52, 0.42)
Moridpour, 2024a (84)	26	RCT	Cinnamon			Type 2 diabetes	FBG	1,755	SMD	−1.32 (−1.77, −0.87)
Moridpour, 2024b (84)	15	RCT	Cinnamon	< 3 g/day		Type 2 diabetes	FBG	1,148	SMD	−0.9 (−1.45, −0.34)
Moridpour, 2024c (84)	11	RCT	Cinnamon	≥3 g/day		Type 2 diabetes	FBG	607	SMD	−1.95 (−2.73, −1.17)
Moridpour, 2024d (84)	19	RCT	Cinnamon			Type 2 diabetes	HbA1c	1,370	SMD	−0.67 (−1.18, −0.15)
Moridpour, 2024e (84)	16	RCT	Cinnamon		≤ 10 weeks	Type 2 diabetes	FBG	813	SMD	−1.70 (−2.48, −0.93)
Moridpour, 2024f (84)	10	RCT	Cinnamon		>10 weeks	Type 2 diabetes	FBG	942	SMD	−1.05 (−1.47, −0.62)
Moridpour, 2024g (84)	12	RCT	Cinnamon	< 3 g/day		Type 2 diabetes	HbA1c	913	SMD	−0.91 (−1.68, −0.13)
Moridpour, 2024h (84)	7	RCT	Cinnamon	≥3 g/day		Type 2 diabetes	HbA1c	457	SMD	−0.29 (−0.76, 0.18)
Moridpour, 2024i (84)	9	RCT	Cinnamon		≤ 10 weeks	Type 2 diabetes	HbA1c	507	SMD	−0.04 (−0.67, 0.59)
Moridpour, 2024j (84)	10	RCT	Cinnamon		>10 weeks	Type 2 diabetes	HbA1c	863	SMD	−1.24 (−1.98, −0.49)
Moridpour, 2024k (84)	8	RCT	Cinnamon			Type 2 diabetes	HOMA-IR	792	SMD	−0.04 (−0.77, −0.10)
Akilen, 2012a (34)	5	RCT	Cinnamon			Type 2 diabetes	FBG	265	MD	0.84 (0.66, 1.02)
Akilen, 2012b (34)	5	RCT	Cinnamon			Type 2 diabetes	HbA1c	314	MD	0.09 (0.04, 0.14)
Suksomboon, 2011a (85)	3	RCT	Cinnamon			Type 2 diabetes	HbA1c	182	MD	0.1 (−0.15, 0.35)
Suksomboon, 2011b (85)	3	RCT	Cinnamon			Type 2 diabetes	FBG	182	MD	−1.05 (−9.52, 7.41)
Garza, 2024a (86)	13	RCT	Cinnamon			Type 2 diabetes	FBG	651	MD	−18.67 (−27.24, −10.10)
Garza, 2024b (86)	11	RCT	Cinnamon			Type 2 diabetes	HbA1c	712	MD	−0.12 (−0.25, 0.02)
Xiaomei, 2024a (87)	3	RCT	Cinnamon			PCOS	BW	185	WMD	−0.47 (−0.80, −0.15)
Xiaomei, 2024b (87)	6	RCT	Cinnamon			PCOS	BMI	418	WMD	−1.17 (−2.63, 0.28)
Xiaomei, 2024c (87)	7	RCT	Cinnamon			PCOS	FBG	373	WMD	−7.72 (−12.33, −3.12)
Xiaomei, 2024d (87)	7	RCT	Cinnamon			PCOS	HOMA-IR	310	WMD	−0.29 (−1.63, 1.05)
Xiaomei, 2024e (87)	3	RCT	Cinnamon			PCOS	CHOL	185	WMD	−11.12 (−19.06, −3.18)
Xiaomei, 2024f (87)	3	RCT	Cinnamon			PCOS	LDL	185	WMD	−11.11 (−18.22, −4.00)
Xiaomei, 2024g (87)	3	RCT	Cinnamon			PCOS	TG	185	WMD	−2.58 (−25.26, 20.09)
Xiaomei, 2024h (87)	3	RCT	Cinnamon			PCOS	HDL	185	WMD	2.32 (−0.15, 4.79)
Heydarpour, 2020a (88)	4	RCT	Cinnamon			PCOS	BMI	338	WMD	−1.47 (−4.07, 1.12)
Heydarpour, 2020b (88)	2	RCT	Cinnamon			PCOS	BW	143	WMD	−0.74 (−3.17, 1.69)
Heydarpour, 2020c (88)	3	RCT	Cinnamon			PCOS	FBG	163	WMD	−5.32 (−10.46, −0.17)
Heydarpour, 2020d (88)	4	RCT	Cinnamon			PCOS	HOMA-IR	180	WMD	−0.69 (−1.38, −0.004)
Heshmati, 2021a (89)	5	RCT	Cinnamon			PCOS	HOMA-IR	250	SMD	−0.84 (−1.52, −0.16)
Heshmati, 2021b (89)	2	RCT	Cinnamon			PCOS	FBG	143	SMD	−0.87 (−1.67, 10.06)
Mousavi, 2020a (52)	12	RCT	Cinnamon			Metabolic syndrome	BW	707	WMD	−1.02 (−1.66, −0.38)
Mousavi, 2020b (52)	13	RCT	Cinnamon			Metabolic syndrome	BMI	764	WMD	−0.51 (−0.74, −0.28)

^a^The number of studies and the total sample size included in each meta-analysis.

**Table 2:** Effect estimates, evidence credibility, risk of bias, and heterogeneity assessment in the included meta-analyses.

**Study, year (Ref)**	**Dosage**	**Duration**	**People**	**Outcomes**	**SMD^a^**	***p*-value^b^**	***I*^2^ (95% CI)**	**Q test *p*-value**	**Egger's *p*-value**	**95% PI**	**O**	**E**	**Excess significance bias *p*-value**	**SMD of the largest study**	**Credibility**
Mandal, 2021a (76)			Type 2 diabetes	FBG	−0.34 (−1.12, 0.45)	0.396935	92.2% (86.6%, 95.5%)	< 0.0001	0.676478	(−2.99, 2.31)	3	1.39	0.03075	−0.33 (−0.73, 0.06)	NS
Mandal, 2021b (76)			Type 2 diabetes	HbA1c	0.70 (−0.30, 1.71)	0.17109	95.8% (93.3%, 97.3%)	< 0.0001	0.0174987	(−2.72, 4.12)	4	1.26	0.001194	−0.29 (−0.66, 0.09)	NS
Mandal, 2021c (76)			Type 2 diabetes	TG	−0.37 (−0.90, 0.17)	0.176917	79.7% (55.9%, 90.7%)	0.0002	0.327299	(−2.01, 1.27)	2	2.28	1	−0.50 (−0.90, −0.10)	NS
Mandal, 2021d (76)			Type 2 diabetes	CHOL	−0.34 (−0.77, 0.08)	0.114169	68.5% (25.5%, 86.6%)	0.0073	0.209201	(−1.57, 0.88)	2	0.51	0.2733	−0.16 (−0.56, 0.23)	NS
Mandal, 2021e (76)			Type 2 diabetes	LDL	0.04 (−0.20, 0.28)	0.754076	12.8% (0.0%, 77.9%)	0.3326	0.280067	(−0.38, 0.46)	0	0.33	0.2733	0.33 (−0.07, 0.73)	NS
Mandal, 2021f (76)			Type 2 diabetes	HDL	0.31 (−0.22, 0.85)	0.251289	75.0% (30.7%, 91.0%)	0.0074	0.31093	(−1.4, 2.04)	1	3.56	2.20E-16	0.99 (0.58, 1.41)	NS
Baker, 2008a (77)			Diabetes	HbA1c	−0.33 (−0.65, 0.01)	0.0424795	23.7% (0.0%, 88.3%)	0.269	0.74791	(−1.06, 0.40)	2	0.21	2.20E-16	−0.04 (−0.52, 0.45)	Weak
Baker, 2008b (77)			Diabetes	FBG	−0.92 (−1.60, 0.24)	0.00853472	74.1% (27.5%, 90.7%)	0.009	0.559087	(−3.09, 1.26)	3	1.32	0.02092	−0.52 (−1.01, −0.02)	Weak
Baker, 2008c (77)			Diabetes	CHOL	−0.69 (−1.28, 0.10)	0.0210771	66.2% (0.9%, 88.5%)	0.031	0.505176	(−2.48, 1.10)	3	1.37	0.02092	−0.52 (−1.01, −0.02)	Weak
Baker, 2008d (77)			Diabetes	TG	−0.53 (−1.41, 0.35)	0.238316	84.8% (62.3%, 93.9%)	0.0002	0.226995	(−3.48, 2.41)	1	0.7	1	−0.33 (−0.82, 0.16)	NS
Baker, 2008e (77)			Diabetes	HDL	−0.45 (−1.59, 0.69)	0.437021	90.4% (74.6%, 96.4%)	< 0.0001	0.86077	(−5.26, 4.36)	1	0.32	2.20E-16	0.21 (−0.28, 0.70)	NS
Baker, 2008f (77)			Diabetes	LDL	0.06 (−0.24, 0.37)	0.689927	0.0% (0.0%, 84.7%)	0.709	0.294348	(−0.43, 0.56)	0	0.21	NA	0.06 (−0.43, 0.54)	NS
Baker, 2008g (77)			Type 2 diabetes	HbA1c	−0.25 (−0.66, 0.17)	0.240397	34.4% (0.0%, 78.6%)	0.2178	0.86576	(−1.55, 1.05)	1	0.16	2.20E-16	−0.04 (−0.52, 0.45)	NS
Leach, 2012a (65)			Diabetes	FBG	−0.75 (−1.18, −0.31)	0.00078021	63.7% (18.0%, 84.0%)	0.0111	0.225909	(−1.98, 0.49)	4	2.29	0.09426	−0.52 (−1.01, 0.02)	Weak
Leach, 2012b (65)			Diabetes	Adverse events	0.83 (0.22, 3.07)	0.77181624	0.0% (0.0%, 85.0%)	0.7149	0.733	(0.05, 14.74)	NA	0.2	NA	1.04 (0.16, 6.86)	NS
Leach, 2012c (65)		≥12 weeks	Diabetes	HbA1c	−0.22 (−0.56, 0.11)	0.19311	58.2% (0.0%, 84.5%)	0.0482	0.945922	(−1.16, 0.71)	2	1.06	0.2636	−0.29 (−0.66, 0.09)	NS
Leach, 2012e (65)	≤ 1 g/day		Diabetes	FBG	−1.12 (−2.08, −0.16)	0.0218219	80.2% (37.5%, 93.7%)	0.0064	0.593327	(−4.95, 2.71)	2	2.5	2.20E-16	−1.24 (−1.82, −0.66)	Weak
Leach, 2012f (65)	1.5–2 g/day		Diabetes	FBG	−0.49 (−1.04, 0.07)	0.0852015	35.0% (0.0%, 79.0%)	0.2146	0.54711	(−2.25, 1.28)	1	0.58	1	−0.40 (−0.92, 0.12)	NS
Leach, 2012g (65)	3 g/day		Diabetes	FBG	−1.19 (−2.68, 0.29)	0.115223	83.2% (29.8%, 96.0%)	0.0148	NA	(−16.98, 14.59)	2	0.63	0.1573	−0.52 (−1.01, 0.02)	NS
Leach, 2012h (65)		< 12 weeks	Diabetes	FBG	−0.88 (−1.75, −0.001)	0.0498671	73.5% (25.7%, 90.6%)	0.0101	0.122904	(−3.67, 1.91)	2	0.34	2.20E-16	−0.25 (−0.87, 0.38)	Weak
Leach, 2012i (65)		≥12 weeks	Diabetes	FBG	−0.70 (−1.19, −0.21)	0.0048341	61.3% (0.0%, 89.0%)	0.0757	0.322077	(−2.51, 1.11)	2	1.42	0.2207	−0.52 (−1.01, 0.02)	Weak
Leach, 2012j (65)	1 g/day		Diabetes	HbA1c	−0.18 (−0.71, 0.35)	0.506545	73.4% (10.7%, 92.1%)	0.0234	0.872787	(−2.27, 1.90)	1	0.67	1	−0.29 (−0.66, 0.09)	NS
Leach, 2012k (65)			Type 2 diabetes	HbA1c	−0.35 (−0.59, −0.10)	0.00528405	8.6% (0.0%, 86.0%)	0.3499	0.554582	(−0.81, 0.11)	2	0.88	0.2482	−0.29 (−0.66, 0.09)	Weak
Zhou, 2022a (78)			Diabetes	FBG	−0.34 (−0.70, 0.03)	0.0694517	85.6% (77.5%, 90.8%)	< 0.0001	0.386402	(−1.76, 1.09)	6	7.68	0.2801	−0.58 (−0.92, 0.24)	NS
Zhou, 2022b (78)			Diabetes	BMI	−0.35 (−1.14, 0.44)	0.386571	94.1% (90.2%, 96.4%)	< 0.0001	0.265369	(−3.06, 2.36)	3	7	2.20E-16	−1.98 (−2.38, 1.56)	NS
Zhou, 2022d (78)			Diabetes	HbA1c	−0.03 (−0.48, 0.42)	0.895877	91.2% (87.1%, 93.9%)	< 0.0001	0.00298754	(−1.88, 1.82)	7	7.73	0.6048	−0.56 (−0.90, 0.22)	NS
Zhou, 2022e (78)			Diabetes	HOMA-IR	−3.07 (−5.68, −0.46)	0.0209429	98.7% (98.0%, 99.2%)	< 0.0001	0.255582	(−12.43, 6.29)	2	1.5	0.2482	−0.47 (−0.81, 0.14)	Weak
Zhou, 2022f (78)			Diabetes	CHOL	−0.67 (−1.42, 0.07)	0.0761422	94.9% (92.6%, 96.5%)	< 0.0001	0.1209	(−3.54, 2.19)	5	1.75	0.01902	0.28 (−0.16, 0.72)	NS
Zhou, 2022g (78)			Diabetes	LDL	−0.32 (−0.97, 0.33)	0.332541	93.6% (90.4%, 95.7%)	< 0.0001	0.98687	(−2.79, 2.15)	2	0.61	0.2943	0.06 (−0.38, 0.50)	NS
Zhou, 2022h (78)			Diabetes	HDL	0.22 (−0.55, 0.99)	0.574794	95.2% (93.1%, 96.7%)	< 0.0001	0.797187	(−2.73, 3.17)	2	0.65	0.2943	−0.08 (−0.52, 0.36)	NS
Zhou, 2022i (78)			Diabetes	TG	−0.42 (−0.99, 0.15)	0.148312	91.7% (87.2%, 94.6%)	< 0.0001	0.73144	(−2.56, 1.72)	3	0.57	0.03594	0.04 (−0.40, 0.48)	NS
Wu, 2022a (9)			Metabolic syndrome	CHOL	−0.27 (−0.51, −0.04)	0.0208634	55.3% (14.4%, 76.6%)	0.0105	0.7664	(−0.98, 0.43)	4	1.11	0.001728	−0.16 (−0.54, 0.21)	NS
Wu, 2022b (9)			Metabolic syndrome	TG	−0.27 (−0.46, −0.09)	0.00399387	30.6% (0.0%, 65.0%)	0.1464	0.729353	(−0.71, 0.17)	2	0.62	0.2963	−0.03 (−0.41, 0.34)	NS
Wu, 2022c (9)			Metabolic syndrome	HDL	−0.05 (−0.20, 0.10)	0.531225	0.0% (0.0%, 58.3%)	0.8334	0.136534	(−0.21, 0.12)	1	3.37	0.1824	−0.37 (−0.74, 0.002)	NS
Wu, 2022d (9)			Metabolic syndrome	LDL	−0.21 (−0.45, 0.02)	0.0730344	56.2% (16.4%, 77.0%)	0.0088	0.57726	(−0.93, 0.50)	3	1.00	0.03671	−0.14 (−0.52, 0.23)	NS
Jamali, 2020a (79)			Type 2 diabetes	TG	−0.73 (−1.21, −0.24)	0.00318792	91.0% (87.1%, 93.7%)	< 0.0001	0.271553	(−2.81, 1.36)	7	1.78	0.0001673	−0.21 (−0.61, 0.18)	Weak
Jamali, 2020b (79)			Type 2 diabetes	CHOL	−1.30 (−2.00, −0.60)	0.000258329	95.1% (93.4%, 96.4%)	< 0.0001	0.00766363	(−4.30, 1.70)	9	1.27	2.20E-16	−0.16 (−0.56, 0.23)	Weak
Jamali, 2020c (79)			Type 2 diabetes	LDL	−0.74 (−1.29, −0.19)	0.00826708	92.8% (90.0%, 94.9%)	< 0.0001	0.0982236	(−3.13, 1.65)	6	1.40	2.55E-07	0.17 (−0.23, 0.56)	Weak
Jamali, 2020d (79)			Type 2 diabetes	HDL	0.23 (−0.39, 0.84)	0.469405	94.2% (91.8%, 95.9%)	< 0.0001	0.711472	(−2.32, 2.78)	3	0.99	6.25E-12	0.99 (0.58, 1.41)	NS
Jamali, 2020a (2, 10)			Type 2 diabetes	SBP	−0.73 (−1.37, −0.09)	0.0261047	86.9% (71.7%, 93.9%)	< 0.0001	0.933946	(−2.82, 1.36)	2	4.98	2.20E-16	−1.39 (−1.84, 0.95)	Weak
Jamali, 2020b (2, 10)			Type 2 diabetes	DBP	−0.73 (−1.34, −0.12)	0.0189347	85.6% (68.3%, 93.5%)	< 0.0001	0.829267	(−2.71, 1.25)	3	4.05	0.2636	−0.78 (−1.19, 0.38)	Weak
Jamali, 2020c (2, 10)			Type 2 diabetes	BW	−0.41 (−0.94, 0.11)	0.125067	85.5% (70.3%, 92.9%)	< 0.0001	0.424523	(−2.11, 1.28)	2	5.91	2.20E-16	−1.18 (−1.54, 0.82)	NS
Jamali, 2020d (2, 10)			Type 2 diabetes	BMI	−0.55 (−1.16, 0.08)	0.0849434	90.8% (83.7%, 94.8%)	< 0.0001	0.405726	(−2.66, 1.56)	2	7.00	2.20E-16	−1.97 (−2.38, 1.56)	NS
Jamali, 2020f (2, 10)		3 months	Type 2 diabetes	SBP	−1.05 (−2.17, 0.08)	0.067833	87.6% (51.7%, 96.8%)	0.0046	NA	(−13.14, 11.04)	1	0.78	1	−0.48 (−1.00, 0.03)	NS
Jamali, 2020g (2, 10)		2 months	Type 2 diabetes	SBP	−0.52 (−1.44, 0.40)	0.271197	90.2% (73.9%, 96.3%)	< 0.0001	0.684211	(−4.40, 3.36)	1	2.98	2.20E-16	−1.39 (−1.84, 0.95)	NS
Jamali, 2020h (2, 10)		3 months	Type 2 diabetes	DBP	−0.39 (−0.97, 0.18)	0.175954	58.4% (0.0%, 90.2%)	0.1209	NA	(−5.85, 5.06)	1	0.14	2.20E-16	−0.11 (−0.62, 0.40)	NS
Jamali, 2020i (2, 10)		2 months	Type 2 diabetes	DBP	−0.95 (−1.89, −0.01)	0.0468814	89.6% (72.0%, 96.1%)	< 0.0001	0.937758	(−4.90, 3.00)	2	2.38	1	−0.78 (−1.19, 0.37)	Weak
Jamali, 2020j (2, 10)		3 months	Type 2 diabetes	BW	−0.74 (−1.53, 0.04)	0.0625737	87.9% (66.1%, 95.7%)	0.0003	0.65901	(−4.02, 2.53)	2	2.98	2.20E-16	−1.18 (−1.54, 0.82)	NS
Jamali, 2020k (2, 10)		2 months	Type 2 diabetes	BW	−0.03 (−0.32, 0.27)	0.863605	0.0% (0.0%, 89.6%)	0.4238	0.492992	(−0.68, 0.63)	0	0.15	NA	−0.01 (−0.45, 0.43)	NS
Jamali, 2020l (2, 10)		3 months	Type 2 diabetes	BMI	−0.82 (−1.78, 0.15)	0.096007	93.5% (86.6%, 96.9%)	< 0.0001	0.278675	(−4.23, 2.59)	2	4.00	2.20E-16	−1.97 (−2.38, 1.56)	NS
Jamali, 2020m (2, 10)		2 months	Type 2 diabetes	BMI	−0.13 (−0.43, 0.17)	0.394572	0.0% (0.0%, 89.6%)	0.4685	0.121593	(−0.78, 0.52)	0	0.16	NA	0.05 (−0.39, 0.49)	NS
Jalali, 2020a (80)			Type 2 diabetes	SBP	−0.73 (−1.37, −0.09)	0.0261047	86.9% (71.7%, 93.9%)	< 0.0001	0.933946	(−2.82, 1.36)	2	4.98	2.20E-16	−1.39 (−1.84, 0.95)	Weak
Jalali, 2020b (80)			Type 2 diabetes	DBP	−0.73 (−1.34, −0.12)	0.0189347	85.6% (68.3%, 93.5%)	< 0.0001	0.829267	(−2.71, 1.25)	3	4.05	0.2636	−0.78 (−1.19, −0.37)	Weak
Jalali, 2020c (80)	>1.7 g/day		Type 2 diabetes	SBP	−0.58 (−1.56, 0.41)	0.253573	89.4% (71.3%, 96.1%)	< 0.0001	0.753191	(−4.74, 3.58)	1	0.22	2.20E-16	−0.13 (−0.57, 0.31)	NS
Jalali, 2020d (80)	< 1.7 g/day		Type 2 diabetes	SBP	−0.95 (−1.84, −0.06)	0.0368614	85.5% (41.3%, 96.4%)	0.0088	NA	(−10.47, 8.57)	1	2	2.20E-16	−1.39 (−1.84, 0.95)	Weak
Jalali, 2020e (80)	>1.7 g/day		Type 2 diabetes	DBP	−0.92 (−1.95, 0.10)	0.0778968	89.7% (72.3%, 96.2%)	< 0.0001	0.542867	(−5.24, 3.40)	2	1.95	1	−0.69 (−1.22, 0.16)	NS
Jalali, 2020f (80)	< 1.7 g/day		Type 2 diabetes	DBP	−0.46 (−1.12, 0.20)	0.168956	75.4% (0.0%, 94.4%)	0.0438	NA	(−7.23, 6.31)	1	1.79	2.20E-16	−0.78 (−1.19, 0.37)	NS
Akilen, 2013a (81)			Type 2 and prediabetes	SBP	−0.95 (−1.71, −0.19)	0.0144131	75.9% (20.9%, 92.7%)	0.0156	0.924683	(−3.97, 2.06)	1	0.97	1	−0.48 (−1.00, 0.03)	Weak
Akilen, 2013b (81)			Type 2 and prediabetes	DBP	−0.35 (−0.74, 0.04)	0.0795348	23.1% (0.0%, 92.0%)	0.2726	0.874345	(−1.48, 0.78)	1	0.19	2.20E-16	−0.11 (−0.62, 0.40)	NS
Akilen, 2013c (81)			Type 2 diabetes	SBP	−1.05 (−2.17, 0.08)	0.067833	87.6% (51.7%, 96.8%)	0.0046	NA	(−13.14, 11.04)	1	0.78	1	−0.48 (−1.00, 0.03)	NS
Akilen, 2013d (81)			Type 2 diabetes	DBP	−0.39 (−0.97, 0.18)	0.175954	58.4% (0.0%, 90.2%)	0.1209	NA	(−5.85, 5.06)	1	0.14	2.20E-16	−0.11 (−0.62, 0.40)	NS
Allen, 2013a (11)			Type 2 diabetes	HbA1c	−0.38 (−0.57, −0.20)	4.87E-05	0.0% (0.0%, 67.6%)	0.5081	0.617358	(−0.61, −0.16)	3	1.47	0.03251	−0.29 (−0.67, 0.09)	Weak
Allen, 2013b (11)			Type 2 diabetes	FBG	−0.79 (−1.14, −0.43)	1.46E-05	64.7% (32.7%, 81.5%)	0.0016	0.01713	(−1.90, 0.33)	7	3.3	0.006769	−0.52 (−1.01, 0.02)	Weak
Allen, 2013c (11)			Type 2 diabetes	CHOL	−0.95 (−1.44, −0.46)	0.000151249	80.3% (65.5%, 88.7%)	< 0.0001	0.00152893	(−2.62, 0.72)	7	3.23	0.006769	−0.52 (−1.01, 0.02)	Weak
Allen, 2013d (11)			Type 2 diabetes	LDL	−0.52 (−1.03, −0.02)	0.0421228	82.5% (69.9%, 89.8%)	< 0.0001	0.000438947	(−2.28, 1.23)	4	0.58	0.001653	0.06 (−0.43, 0.54)	Weak
Allen, 2013e (11)			Type 2 diabetes	HDL	−0.22 (−0.64, 0.20)	0.29613	73.5% (46.2%, 87.0%)	0.0004	0.596269	(−1.54, 1.09)	1	0.85	1	0.21 (−0.28, 0.70)	NS
Allen, 2013f (11)			Type 2 diabetes	TG	−0.45 (−0.79, −0.10)	0.0109115	64.1% (31.4%, 81.2%)	0.0019	0.0151028	(−1.519, 0.627	4	1.76	0.1179	−0.33 (−0.82, 0.16)	Weak
Yu, 2023a (82)			Type 2 diabetes	FBG	−0.50 (−0.68, −0.32)	6.11E-08	40.0% (0.0%, 68.8%)	0.0673	0.18256	(−0.99, −0.01)	5	7.71	0.08722	−0.58 (−0.92, 0.24)	Weak
Yu, 2023b (82)			Type 2 diabetes	HbA1c	0.20 (−0.40, 0.81)	0.511236	92.6% (88.9%, 95.0%)	< 0.0001	0.511236	(−2.12, 2.52)	4	9.36	0.0008581	−0.88 (−1.29, 0.46)	NS
Yu, 2023c (82)			Type 2 diabetes	TG	−0.47 (−1.08, 0.14)	0.129593	92.2% (87.7%, 95.0%)	< 0.0001	0.605967	(−2.70, 1.76)	4	4.32	1	−0.50 (−0.90, 0.10)	NS
Yu, 2023d (82)			Type 2 diabetes	CHOL	−0.23 (−0.43, −0.03)	0.0261345	21.3% (0.0%, 62.4%)	0.2535	0.558811	(−0.64, 0.18)	3	0.81	0.03389	−0.16 (−0.56, 0.23)	Weak
Yu, 2023e (82)			Type 2 diabetes	HDL	0.32 (−0.46, 1.10)	0.420332	95.4% (93.4%, 96.8%)	< 0.0001	0.767971	(−2.68, 3.31)	3	9.45	2.11E-13	0.99 (0.58, 1.41)	NS
Yu, 2023f (82)			Type 2 diabetes	LDL	−0.45 (−1.33, 0.43)	0.317201	95.5% (93.0%, 97.1%)	< 0.0001	0.65695	(−3.56, 2.66)	2	1.82	1	0.33 (−0.07, 0.73)	NS
Yu, 2023g (82)	≥1.2 g/day		Type 2 diabetes	FBG	−0.48 (−0.67, −0.28)	1.27E-06	16.9% (0.0%, 59.8%)	0.2968	0.573133	(−0.83, −0.12)	3	4.73	0.1441	−0.58 (−0.92, 0.24)	Weak
Yu, 2023h (82)	< 1.2 g/day		Type 2 diabetes	FBG	−0.52 (−0.89, −0.14)	0.00692176	63.7% (4.2%, 86.2%)	0.0265	0.224066	(−1.60, 0.56)	2	4.69	2.20E-16	−1.00 (−1.36, 0.65)	Weak
Yu, 2023i (82)		≥8 weeks	Type 2 diabetes	FBG	−0.53 (−0.75, −0.32)	1.34E-06	39.0% (0.0%, 73.1%)	0.1189	0.155534	(−1.05, −0.01)	4	5.09	0.4652	−0.58 (−0.92, 0.24)	Weak
Yu, 2023j (82)		< 8 weeks	Type 2 diabetes	FBG	−0.43 (−0.78, −0.08)	0.0163732	49.0% (0.0%, 81.3%)	0.0974	0.862522	(−1.35, 0.49)	1	3.69	0.0007962	−0.79 (−1.20, 0.38)	Weak
De Moura, 2025a (83)			Type 2 diabetes	FBG	−0.61 (−0.91, −0.31)	7.00E-05	85.4% (78.9%, 89.8%)	< 0.0001	0.280333	(−1.96, 0.73)	12	6.75	0.02064	−0.40 (−0.68, 0.12)	Suggestive
De Moura, 2025b (83)	≤ 2 g/day		Type 2 diabetes	FBG	−0.58 (−1.03, −0.14)	0.0105879	90.1% (85.0%, 93.5%)	< 0.0001	0.613233	(−2.30, 1.14)	8	4.46	0.01623	−0.40 (−0.68, 0.12)	Weak
De Moura, 2025c (83)	>2 g/day		Type 2 diabetes	FBG	−0.61 (−0.91, −0.30)	9.27E-05	53.0% (0.0%, 78.9%)	0.0372	0.0787397	(−1.42, 0.21)	4	3.89	1	−0.58 (−0.92, 0.24)	Weak
De Moura, 2025d (83)			Type 2 diabetes	HbA1c	−0.17 (−0.63, 0.29)	0.464073	90.9% (87.0%, 93.6%)	< 0.0001	0.205603	(−2.12, 1.78)	8	7.81	1	−0.56 (−0.90, 0.22)	NS
De Moura, 2025e (83)	≤ 2 g/day		Type 2 diabetes	HbA1c	0.03 (−0.69, 0.74)	0.941697	93.8% (90.8%, 95.9%)	< 0.0001	0.194033	(−2.64, 2.69)	6	8.38	0.1757	−1.03 (−1.38, 0.67)	NS
De Moura, 2025f (83)	>2 g/day		Type 2 diabetes	HbA1c	−0.29 (−0.65, 0.07)	0.112869	58.0% (0.0%, 83.0%)	0.0363	0.862252	(−1.27, 0.68)	2	2.68	0.4142	−0.56 (−0.90, 0.22)	NS
De Moura, 2025g (83)			Type 2 diabetes	HOMA-IR	−2.43 (−4.47, −0.39)	0.0193197	98.3% (97.5%, 98.9%)	< 0.0001	0.158823	(−9.40, 4.54)	1	0.66	1	−0.23 (−0.57, 0.11)	Weak
De Moura, 2025h (83)			Type 2 diabetes	CHOL	−1.32 (−2.02, −0.62)	0.000227381	95.1% (93.4%, 96.4%)	< 0.0001	0.0120453	(−4.32, 1.69)	8	1.32	4.85E-13	−0.16 (−0.54, 0.21)	Weak
De Moura, 2025i (83)	≤ 2 g/day		Type 2 diabetes	CHOL	−1.38 (−2.30, −0.44)	0.00378103	96.2% (94.6%, 97.3%)	< 0.0001	0.0661536	(−4.93, 2.18)	5	0.92	2.73E-05	−0.16 (−0.54, 0.21)	Weak
De Moura, 2025j (83)	>2 g/day		Type 2 diabetes	CHOL	−1.11 (−2.12, −0.10)	0.0315592	90.1% (79.9%, 95.2%)	< 0.0001	0.0269077	(−4.34, 2.13)	3	0.70	0.02535	0.28 (−0.16, 0.72)	Weak
De Moura, 2025k (83)			Type 2 diabetes	HDL	0.38 (−0.20, 0.97)	0.200921	93.5% (90.6%, 95.5%)	< 0.0001	0.920665	(−1.96, 2.72)	2	0.86	0.298	−0.10 (−0.48, 0.27)	NS
De Moura, 2025l (83)	≤ 2 g/day		Type 2 diabetes	HDL	0.52 (−0.23, 1.27)	0.176736	94.8% (92.2%, 96.5%)	< 0.0001	0.905938	(−2.28, 3.32)	2	0.65	0.2918	−0.10 (−0.48, 0.27)	NS
De Moura, 2025m (83)	>2 g/day		Type 2 diabetes	HDL	−0.04 (−0.35, 0.27)	0.790324	8.1% (0.0%, 90.4%)	0.337	0.576594	(−0.79, 0.71)	0	0.19	NA	−0.08 (−0.52, 0.36)	NS
De Moura, 2025n (83)			Type 2 diabetes	LDL	−0.88 (−1.48, −0.29)	0.00370783	93.3% (90.4%, 95.2%)	< 0.0001	0.0993596	(−3.35, 1.59)	5	1.15	3.47E-05	−0.14 (−0.52, 0.23)	Weak
De Moura, 2025o (83)	≤ 2 g/day		Type 2 diabetes	LDL	−0.84 (−1.63, −0.05)	0.0381273	4.7% (92.1%, 96.4%)	< 0.0001	0.408986	(−3.76, 2.09)	3	0.79	0.03501	−0.14 (−0.52, 0.23)	Weak
De Moura, 2025p (83)	>2 g/day		Type 2 diabetes	LDL	−0.91 (−1.79, −0.02)	0.0460369	87.7% (73.9%, 94.3%)	< 0.0001	0.00228121	(−3.72, 1.91)	2	0.27	2.20E-16	0.06 (−0.38, 0.50)	Weak
De Moura, 2025q (83)			Type 2 diabetes	TG	−0.82 (−1.30, −0.34)	0.000903303	90.8% (86.7%, 93.6%)	< 0.0001	0.249792	(−2.86, 1.22)	8	0.82	4.85E-13	−0.03 (−0.41, 0.34)	Weak
De Moura, 2025r (83)	≤ 2 g/day		Type 2 diabetes	TG	−0.89 (−1.56, −0.23)	0.0084419	93.3% (89.8%, 95.5%)	< 0.0001	0.473669	(−3.39, 1.61)	5	0.57	2.73E-05	−0.03 (−0.41, 0.34)	Weak
De Moura, 2025s (83)	>2 g/day		Type 2 diabetes	TG	−0.58 (−1.07, −0.10)	0.0176497	63.2% (2.7%, 86.1%)	0.0281	0.0134925	(−1.95, 0.78)	3	0.36	2.20E-16	0.04 (−0.40, 0.48)	Weak
De Moura, 2025t (83)			Type 2 diabetes	BMI	−0.46 (−1.01, 0.08)	0.0962971	91.0% (84.6%, 94.7%)	< 0.0001	0.860008	(−2.35, 1.43)	2	0.40	2.20E-16	0.00 (−0.34, 0.34)	NS
De Moura, 2025u (83)	≤ 2 g/day		Type 2 diabetes	BMI	−0.67 (−1.48, 0.14)	0.103368	93.8% (88.4%, 96.7%)	< 0.0001	0.836494	(−3.40, 2.06)	2	0.25	2.20E-16	0.00 (−0.34, 0.34)	NS
De Moura, 2025v (83)	>2 g/day		Type 2 diabetes	BMI	−0.05 (−0.33, 0.23)	0.739684	0.0% (0.0%, 89.6%)	0.4308	0.0232571	(−0.67, 0.57)	0	0.16	NA	0.03 (−0.41, 0.47)	NS
De Moura, 2025w (83)			Type 2 diabetes	BW	−0.42 (−0.93, 0.09)	0.1062	85.4% (70.2%, 92.9%)	< 0.0001	0.569089	(−2.07, 1.23)	2	5.91	2.20E-16	−1.18 (−1.54, 0.82)	NS
De Moura, 2025x (83)	≤ 2 g/day		Type 2 diabetes	BW	−0.71 (−1.56, 0.13)	0.0980594	89.6% (72.1%, 96.2%)	< 0.0001	0.670642	(−4.26, 2.84)	2	2.98	2.20E-16	−1.18 (−1.54, 0.82)	NS
De Moura, 2025y (83)	>2 g/day		Type 2 diabetes	BW	−0.08 (−0.36, 0.20)	0.592343	0.0% (0.0%, 89.6%)	0.5927	0.00468611	(−0.69, 0.54)	0	0.15	NA	−0.01 (−0.45, 0.44)	NS
Namazi, 2019a (51)			Type 2 diabetes	FBG	−0.75 (−1.11, −0.40)	3.33E-05	87.4% (82.0%, 91.2%)	< 0.0001	0.287079	(−2.34, 0.83)	13	5.52	0.0006363	−0.40 (−0.74, 0.06)	Suggestive
Namazi, 2019b (51)			Type 2 diabetes	HbA1c	0.09 (−0.44, 0.62)	0.736994	92.0% (88.1%, 94.6%)	< 0.0001	0.010009	(−1.99, 2.18)	6	4.23	0.2294	−0.41 (−0.75, 0.07)	NS
Namazi, 2019c (51)			Type 2 diabetes	BW	−0.32 (−0.87, 0.24)	0.260331	76.9% (37.1%, 91.5%)	0.0046	0.493122	(−2.13, 1.49)	1	0.2	2.20E-16	−0.01 (−0.45, 0.43)	NS
Namazi, 2019d (51)			Type 2 diabetes	BMI	−0.34 (−0.73, 0.05)	0.0913242	64.3% (6.2%, 86.4%)	0.0243	0.28566	(−1.48, 0.80)	1	0.26	2.20E-16	0.03 (−0.41, 0.47)	NS
Moridpour, 2024a (84)			Type 2 diabetes	FBG	−0.60 (−0.87, −0.34)	7.64E-06	84.0% (77.3%, 88.7%)	< 0.0001	0.41989	(−1.85, 0.64)	14	7.46	0.001669	−0.40 (−0.68, 0.12)	Suggestive
Moridpour, 2024b (84)	< 3 g/day		Type 2 diabetes	FBG	−0.47 (−0.83, −0.11)	0.0111989	87.1% (80.1%, 91.7%)	< 0.0001	0.847686	(−1.88, 0.95)	9	4.83	0.02567	−0.40 (−0.68, 0.12)	Weak
Moridpour, 2024c (84)	≥3 g/day		Type 2 diabetes	FBG	−0.80 (−1.20, −0.41)	6.82E-05	77.9% (59.6%, 87.9%)	< 0.0001	0.102421	(−2.12, 0.51)	5	4.55	1	−0.58 (−0.92, 0.24)	Weak
Moridpour, 2024d (84)			Type 2 diabetes	HbA1c	−0.08 (−0.47, 0.31)	0.67516	90.4% (86.2%, 93.3%)	< 0.0001	0.00226259	(−1.74, 1.58)	7	9.57	0.1393	−0.56 (−0.90, 0.22)	NS
Moridpour, 2024e (84)		≤ 10 weeks	Type 2 diabetes	FBG	−0.74 (−1.27, −0.22)	0.00561674	90.0% (85.1%, 93.4%)	< 0.0001	0.390416	(−2.85, 1.36)	8	8.50	0.577	−0.79 (−1.20, 0.38)	Weak
Moridpour, 2024f (84)		>10 weeks	Type 2 diabetes	FBG	−0.50 (−0.66, −0.34)	1.37E-09	29.9% (0.0%, 66.5%)	0.17	0.632093	(−0.87, −0.13)	6	4.44	0.1967	−0.79 (−1.20, −0.38)	Weak
Moridpour, 2024g (84)	< 3 g/day		Type 2 diabetes	HbA1c	0.06 (−0.53, 0.65)	0.84925	93.5% (90.3%, 95.7%)	< 0.0001	0.0108781	(−2.14, 2.254)	6	3.97	0.21	−0.41 (−0.75, 0.07)	NS
Moridpour, 2024h (84)	≥3 g/day		Type 2 diabetes	HbA1c	−0.19 (−0.45, 0.07)	0.161052	37.1% (0.0%, 75.0%)	0.1588	0.184908	(−0.79, 0.42)	1	3.36	0.1025	−0.56 (−0.90, 0.22)	NS
Moridpour, 2024i (84)		≤ 10 weeks	Type 2 diabetes	HbA1c	0.99 (−0.15, 2.13)	0.0892662	95.8% (93.3%, 97.3%)	< 0.0001	0.0151	(−2.90, 4.87)	2	4.83	0.01207	−0.88 (−1.29, 0.46)	NS
Moridpour, 2024j (84)		>10 weeks	Type 2 diabetes	HbA1c	−0.48 (−0.67, −0.28)	1.20E-06	46.4% (0.0%, 74.2%)	0.052	0.617071	(−1.00, 0.04)	5	6.59	0.1675	−0.56 (−0.90, 0.22)	Weak
Moridpour, 2024k (84)			Type 2 diabetes	HOMA-IR	−1.76 (−2.86, −0.67)	0.00159355	97.5% (96.3%, 98.3%)	< 0.0001	0.0650981	(−5.53, 2.01)	3	0.96	0.03075	−0.19 (−0.47, 0.09)	Weak
Akilen, 2012a (34)			Type 2 diabetes	FBG	−0.93 (−1.64, −0.21)	0.0109321	86.2% (69.9%, 93.7%)	< 0.0001	0.668263	(−3.25, 1.39)	3	1.99	0.3613	−0.52 (−1.01, 0.02)	Weak
Akilen, 2012b (34)			Type 2 diabetes	HbA1c	−0.36 (−0.58, −0.14)	0.00169592	0.4% (0.0%, 79.3%)	0.4039	0.653098	(−0.68, −0.04)	3	1.70	0.3613	−0.41 (−0.78, 0.03)	Weak
Suksomboon, 2011a (85)			Type 2 diabetes	HbA1c	−0.32 (−0.90, 0.26)	0.274578	61.0% (0.0%, 91.0%)	0.1091	NA	(−5.90, 5.26)	1	0.10	2.20E-16	−0.04 (−0.52, 0.45)	NS
Suksomboon, 2011b (85)			Type 2 diabetes	FBG	−0.86 (−1.56, −0.16)	0.0162166	71.2% (0.0%, 93.5%)	0.0622	NA	(−7.97, 6.24)	2	0.94	0.1573	−0.52 (−1.01, 0.02)	Weak
Garza, 2024a (86)			Type 2 diabetes	FBG	−0.65 (−0.93, −0.37)	6.03E-06	63.0% (32.7%, 79.7%)	0.0012	0.0082095	(−1.55, 0.26)	6	5.65	1	−0.58 (−0.92, 0.24)	Weak
Garza, 2024b (86)			Type 2 diabetes	HbA1c	−0.29 (−0.47, −0.11)	0.00130262	24.9% (0.0%, 62.7%)	0.2061	0.302507	(−0.67, 0.10)	4	6.04	0.2259	−0.56 (−0.90, 0.22)	Weak
Xiaomei, 2024a (87)			PCOS	BW	−0.07 (−0.36, 0.22)	0.632996	0.0% (0.0%, 89.6%)	0.9815	0.214965	(−0.70, 0.56)	0	0.16	NA	−0.04 (−0.47, 0.39)	NS
Xiaomei, 2024b (87)			PCOS	BMI	−0.30 (−0.85, 0.24)	0.27657	84.9% (69.0%, 92.7%)	< 0.0001	0.104852	(−2.04, 1.43)	1	5.75	2.20E-16	−1.28 (−1.60, 0.95)	NS
Xiaomei, 2024c (87)			PCOS	FBG	−0.71 (−1.22, −0.20)	0.00618153	80.5% (60.5%, 90.4%)	< 0.0001	0.864442	(−2.32, 0.89)	3	6.21	0.001194	−1.17 (−1.59, 0.74)	Weak
Xiaomei, 2024d (87)			PCOS	HOMA-IR	−1.18 (−2.29, −0.08)	0.0356051	93.9% (89.4%, 96.5%)	< 0.0001	0.448691	(−4.89, 2.52)	3	2.73	1	−0.67 (−1.11, 0.23)	Weak
Xiaomei, 2024e (87)			PCOS	CHOL	−0.33 (−0.62, −0.04)	0.025388	0.0% (0.0%, 89.6%)	0.6857	0.527863	(−0.97, 0.31)	0	0.87	0.2207	−0.37 (−0.80, 0.06)	Weak
Xiaomei, 2024f (87)			PCOS	LDL	−0.43 (−0.72, −0.14)	0.00386824	0.0% (0.0%, 89.6%)	0.454	0.618524	(−1.07, 0.21)	2	1.24	0.2207	−0.46 (−0.90, 0.03)	Weak
Xiaomei, 2024g (87)			PCOS	TG	−0.02 (−0.50, 0.45)	0.923332	61.0% (0.0%, 88.9%)	0.0772	0.375327	(−1.78, 1.73)	0	0.42	NA	−0.23 (−0.66, 0.20)	NS
Xiaomei, 2024h (87)			PCOS	HDL	0.22 (−0.20, 0.64)	0.295191	50.0% (0.0%, 85.5%)	0.1353	0.265094	(−1.23, 1.68)	1	1.81	0.2207	0.59 (0.15, 1.03)	NS
Heydarpour, 2020a (88)			PCOS	BMI	−0.41 (−1.15, 0.33)	0.277183	89.2% (75.2%, 95.3%)	< 0.0001	0.342701	(−2.93, 2.12)	1	3.80	2.20E−16	−1.28 (−1.60, 0.95)	NS
Heydarpour, 2020b (88)			PCOS	BW	−0.06 (−0.39, 0.27)	0.718084	0.00%	0.8821	NA	(−2.19, 2.07)	0	0.11	NA	−0.04 (−0.47, 0.39)	NS
Heydarpour, 2020c (88)			PCOS	FBG	−0.62 (−0.93, −0.30)	0.000122594	0.0% (0.0%, 89.6%)	0.6313	0.669266	(−1.31, 0.07)	1	2.12	0.2207	−0.77 (−1.21, 0.33)	Weak
Heydarpour, 2020d (88)			PCOS	HOMA-IR	−0.56 (−0.86, −0.26)	0.000243851	0.0% (0.0%, 84.7%)	0.6928	0.0279056	(−1.05, −0.07)	2	1.96	1	−0.67 (−1.11, 0.23)	Weak
Heshmati, 2021a (89)			PCOS	HOMA-IR	−1.58 (−3.25, 0.10)	0.0645241	96.2% (92.8%, 97.9%)	< 0.0001	0.466463	(−7.52, 4.37)	3	2.05	0.3173	−0.67 (−1.11, 0.23)	NS
Heshmati, 2021b (89)			PCOS	FBG	−0.63 (−0.97, −0.30)	0.000226792	0.00%	0.3565	NA	(−2.82, 1.55)	1	1.73	2.20E-16	−0.77 (−1.21, 0.33)	Weak
Mousavi, 2020a (52)			Metabolic syndrome	BW	−0.35 (−0.73, 0.03)	0.0725916	81.1% (63.8%, 90.2%)	< 0.0001	0.532764	(−1.60, 0.90)	2	7.90	2.20E-16	−1.18 (−1.54, 0.82)	NS
Mousavi, 2020b (52)			Metabolic syndrome	BMI	−0.33 (−0.91, 0.25)	0.266115	92.2% (87.4%, 95.2%)	< 0.0001	0.446046	(−2.40, 1.74)	3	1.32	0.03389	−0.22 (−0.59, 0.14)	NS

^a^SMD of random-effects model.

^b^p value of random-effects model.

O, the observed number of studies; E, the expected number; NS, not-significant.

### 3.1 Cinnamon and FBG outcomes

**Figure 3:**
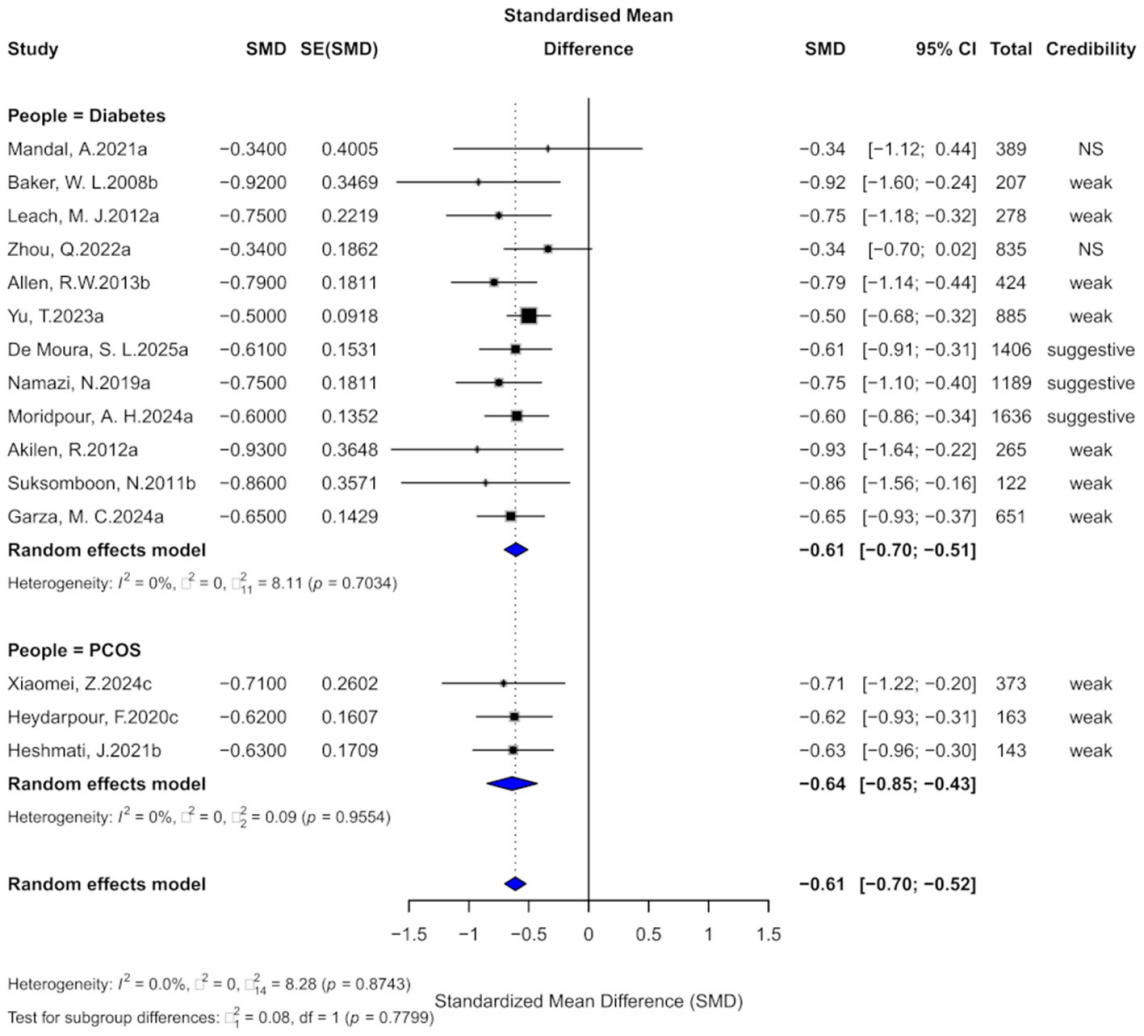
Forest plot of the effect of cinnamon supplementation on FBG in patients with metabolic diseases.

Fifteen comparisons evaluated the effect of cinnamon supplementation on FBG in patients with metabolic diseases. The pooled analysis showed a significant reduction (SMD = −0.61, 95% CI: −0.70 to −0.52) with no heterogeneity (*I*^2^ = 0%, *p* = 0.8743), suggesting high consistency (Figure 3). However, 13 comparisons (86.7%) exhibited substantial heterogeneity (*I*^2^ > 50%), possibly due to variation in dosage, intervention duration, and population characteristics. Egger's test indicated marginal funnel plot asymmetry (bias = −1.00, *p* = 0.051), and two comparisons showed small-study effects (*p* < 0.05). Excess significance bias was observed in 10 comparisons (66.7%). After imputing four missing studies via the trim-and-fill method, the effect remained significant (SMD = −0.58, 95% CI: −0.66 to −0.51), supporting the robustness of the findings.

In terms of evidence strength, three comparisons (20%) were rated as “suggestive,” 10 (66.7%) as “weak,” and two (13.3%) as “non-significant.” Only one comparison had a 95% prediction interval excluding the null. At *p* < 0.05, 93.3% were significant, but only one remained significant at *p* < 0.000001.

Subgroup analysis showed consistent effects in diabetes (SMD = −0.61, 95% CI: −0.70 to −0.51) and PCOS (SMD = −0.64, 95% CI: −0.85 to −0.43), with no significant subgroup difference (χ^2^ = 0.08, *p* = 0.7799).

### 3.2 Cinnamon and HbA1c outcomes

**Figure 4:**
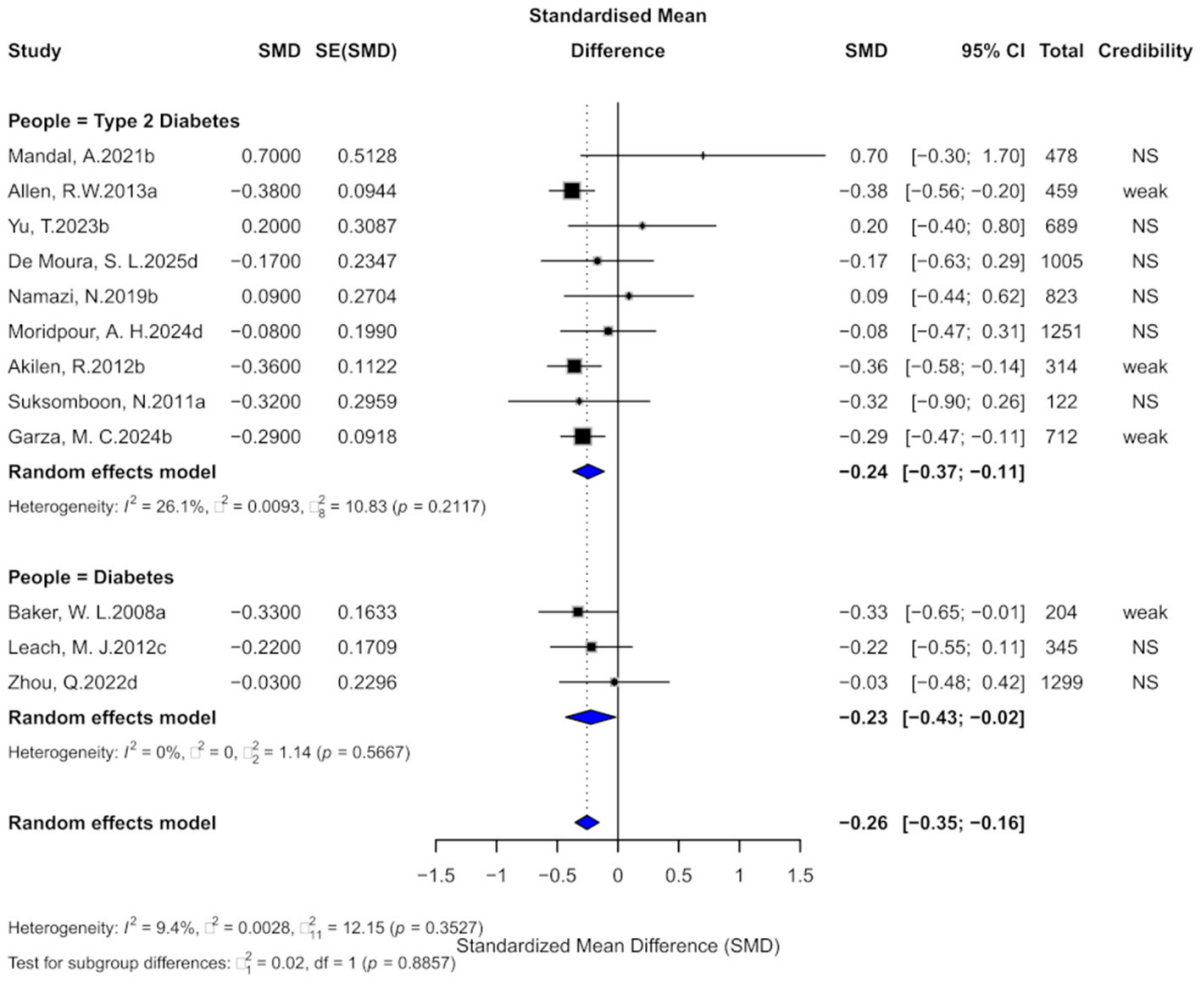
Forest plot of the effect of cinnamon supplementation on HbA1c in patients with metabolic diseases.

Twelve comparisons assessed the impact of cinnamon on HbA1c levels. Cinnamon supplementation was associated with a moderate reduction (SMD = −0.26, 95% CI: −0.35 to −0.16), with low heterogeneity (*I*^2^ = 9.4%, *p* = 0.3527; Figure 4). Nevertheless, eight comparisons (66.7%) showed substantial heterogeneity, and 4 (33.3%) showed small-study effects. Egger's test confirmed funnel plot asymmetry (*p* = 0.0006), and excess significance was observed in five comparisons. After adding 5 imputed studies, the adjusted effect remained significant (SMD = −0.32, 95% CI: −0.44 to −0.21), though heterogeneity increased moderately (*I*^2^ = 38.1%, *p* = 0.0565), suggesting stable yet cautious interpretation.

Evidence grading showed four comparisons (33.3%) with “weak” evidence, and 8 (66.7%) as “non-significant.” Only two comparisons had prediction intervals excluding the null. Statistically significant results were found in 33.3% at *p* < 0.05, but only 1 comparison remained significant at *p* < 0.001.

Subgroup analysis indicated similar reductions in HbA1c among diabetes (SMD = −0.23) and type 2 diabetes (SMD = −0.24), with no significant subgroup difference (χ^2^ = 0.02, *p* = 0.8857).

### 3.3 Cinnamon and HOMA-IR outcomes

**Figure 5:**
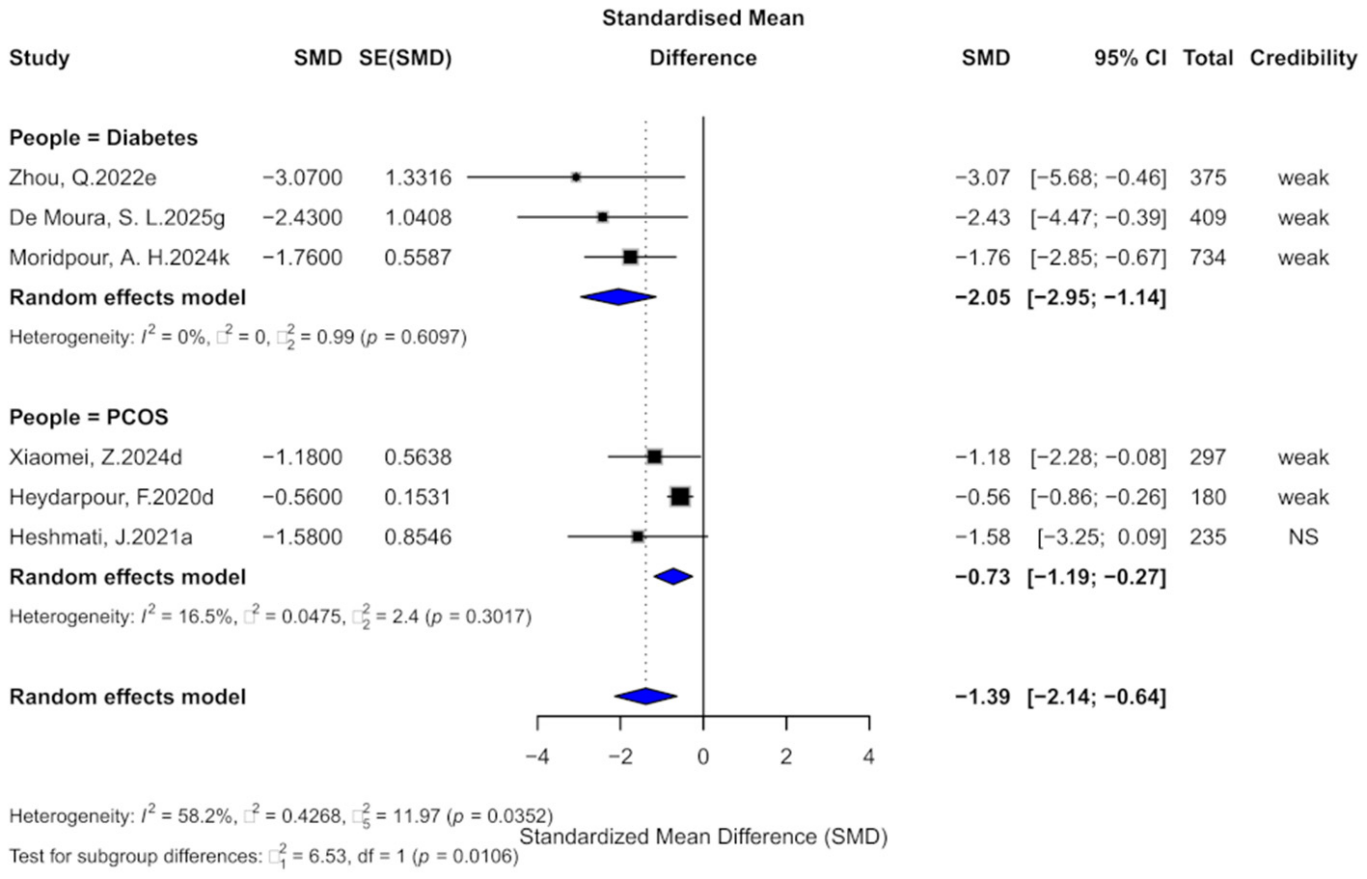
Forest plot of the effect of cinnamon supplementation on HOMA-IR in patients with metabolic diseases.

Six comparisons examined the effect of cinnamon on HOMA-IR. The pooled effect was significant (SMD = −1.39, 95% CI: −2.14 to −0.64) with high heterogeneity (*I*^2^ = 58.2%, *p* = 0.0352; Figure 5). Egger's test indicated small-study effects (*p* < 0.05), and one comparison suggested excess significance bias. After trim-and-fill adjustment (four studies imputed), the effect attenuated (SMD = −0.79, 95% CI: −1.89 to 0.30), with the wide confidence interval crossing the null, indicating reduced certainty and the need for cautious interpretation.

In terms of evidence, five comparisons (83.3%) provided “weak” and one “non-significant” evidence. Only one had a prediction interval excluding zero. Five comparisons were significant at *p* < 0.05, but only one at *p* < 0.001.

Subgroup analysis revealed stronger effects in diabetes (SMD = −2.05, 95% CI: −2.95 to −1.14) than in PCOS (SMD = −0.73, 95% CI: −1.19 to −0.27), with a significant subgroup difference (χ^2^ = 6.53, *p* = 0.0106).

### 3.4 Cinnamon and TG outcomes

**Figure 6:**
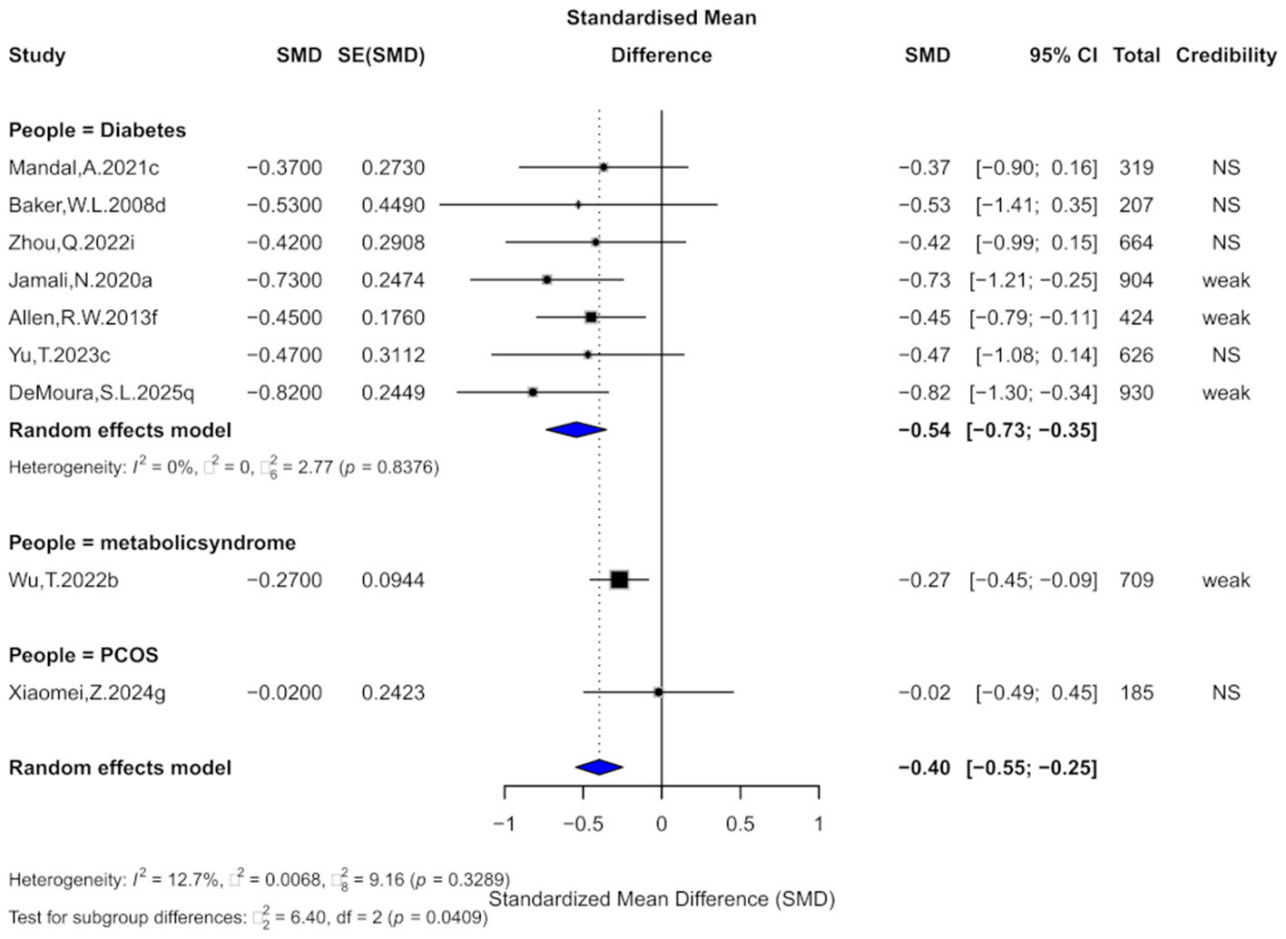
Forest plot of the effect of cinnamon supplementation on TG in patients with metabolic diseases.

Nine comparisons evaluated the effect of cinnamon on TG levels. The pooled analysis showed a significant reduction (SMD = −0.40, 95% CI: −0.55 to −0.25) with low heterogeneity (*I*^2^ = 12.7%; Figure 6). However, 88.9% of comparisons had high within-study heterogeneity. Egger's test showed no significant bias (bias = −1.03, *p* = 0.212), though one comparison had small-study effects and three showed excess significance bias. Trim-and-fill imputation of three studies slightly reduced the effect size (SMD = −0.31, 95% CI: −0.52 to −0.11), with moderate heterogeneity (*I*^2^ = 38.9%).

Regarding evidence strength, 44.4% were rated “weak,” and the rest “non-significant.” No comparisons reached “suggestive” level, and all prediction intervals included the null. Only 44.4% were significant at *p* < 0.05, just one remained significant at *p* < 0.001.

Subgroup analysis showed the greatest TG reduction in diabetes (SMD = −0.54), followed by metabolic syndrome (SMD = −0.27), with no effect in PCOS (SMD = −0.02). Subgroup differences were significant (χ^2^ = 6.4, *p* = 0.0409), suggesting population-dependent effects.

### 3.5 Cinnamon and CHOL outcomes

**Figure 7:**
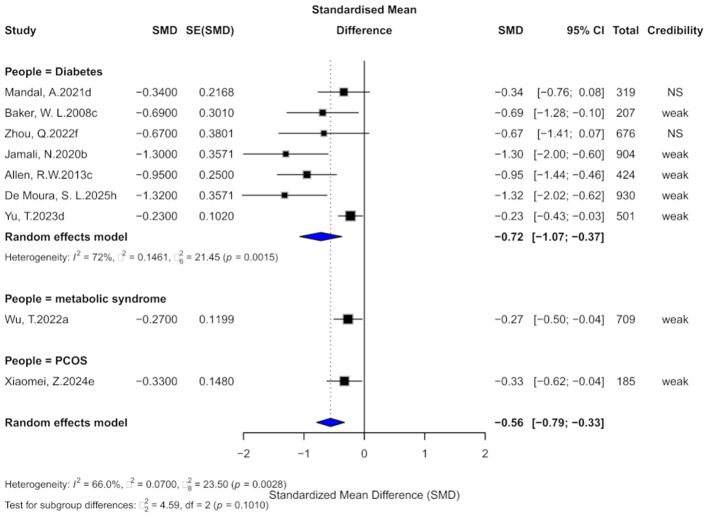
Forest plot of the effect of cinnamon supplementation on CHOL in patients with metabolic diseases.

Nine comparisons evaluated the effect of cinnamon supplementation on CHOL levels. Pooled analysis demonstrated a significant reduction in CHOL (SMD = −0.56, 95% CI: −0.79 to −0.33), although heterogeneity was substantial (*I*^2^ = 66.6%; Figure 7). Notably, 77.8% of comparisons exhibited considerable within-study heterogeneity (*I*^2^ > 50%). Three comparisons showed evidence of small-study effects, and four indicated excess significance bias. Egger's test revealed significant publication bias (bias = −3.18, *p* = 0.002). After imputing four potentially missing studies using the trim-and-fill method, the effect size slightly attenuated (SMD = −0.31, 95% CI: −0.70 to 0.08), and heterogeneity increased slightly (*I*^2^ = 77.1%).

Regarding the strength of evidence, 77.8% of comparisons were rated as “weak,” and the remaining as “non-significant,” with none reaching the level of “suggestive” or higher. All 95% prediction intervals included the null value. In terms of statistical significance, 77.8% of comparisons achieved *p* < 0.05, and three remained significant at the stricter threshold of *p* < 0.001.

Subgroup analyses showed beneficial effects across diabetic (SMD = −0.72), metabolic syndrome (SMD = −0.27), and PCOS populations (SMD = −0.33), with no significant difference between subgroups (χ^2^ = 4.59, *p* = 0.1010).

### 3.6 Cinnamon and HDL outcomes

**Figure 8:**
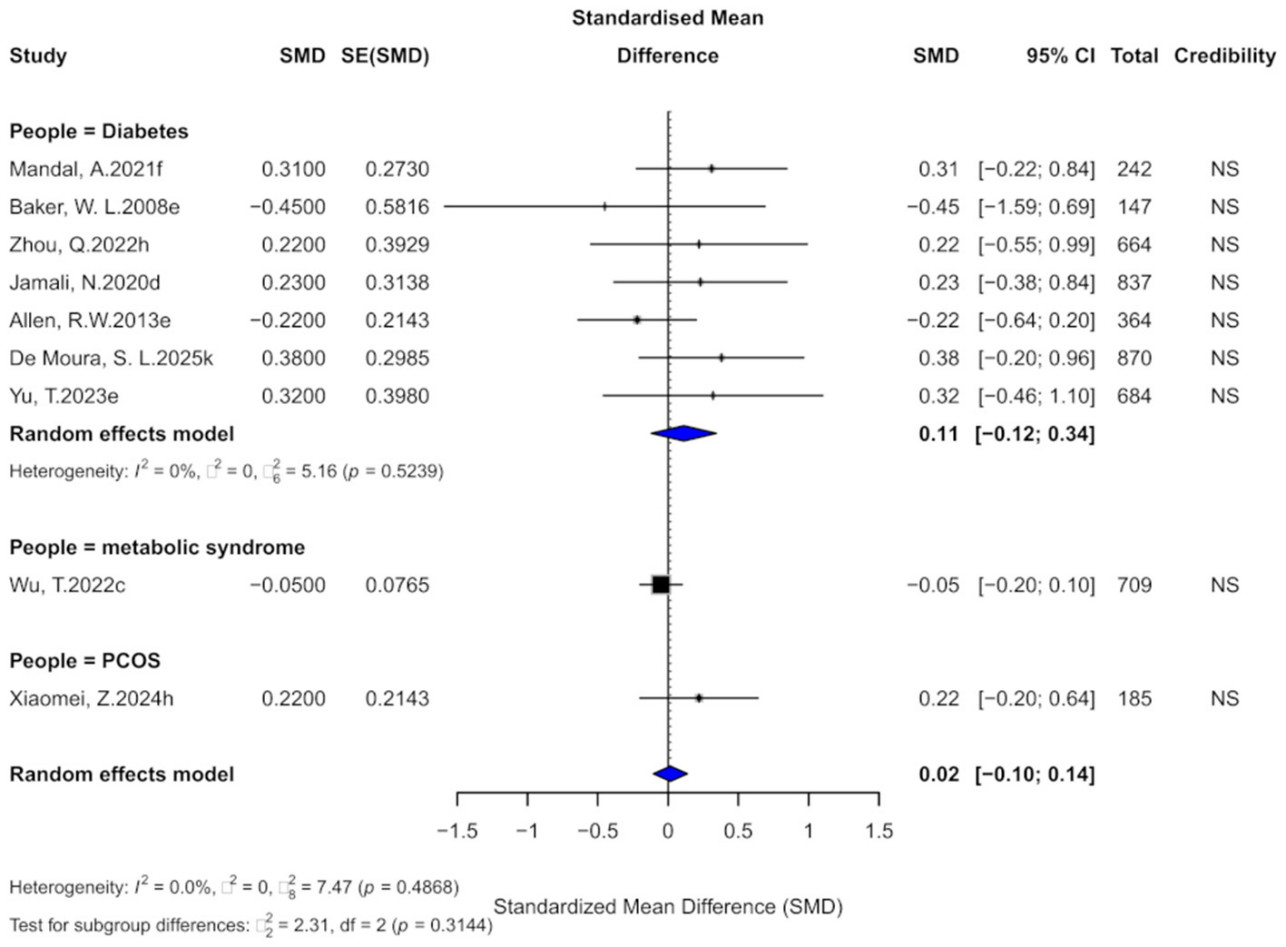
Forest plot of the effect of cinnamon supplementation on HDL in patients with metabolic diseases.

Nine comparisons evaluated the effect of cinnamon supplementation on HDL levels. The pooled analysis showed a negligible and non-significant effect (SMD = 0.02, 95% CI: −0.10 to 0.14), with no observed heterogeneity across studies (*I*^2^ = 0%; Figure 8). The current evidence does not support a significant impact of cinnamon supplementation on HDL levels in patients with metabolic diseases. In terms of evidence grading, all comparisons were classified as “non-significant,” with none reaching the “weak” or higher level.

### 3.7 Cinnamon and LDL outcomes

**Figure 9:**
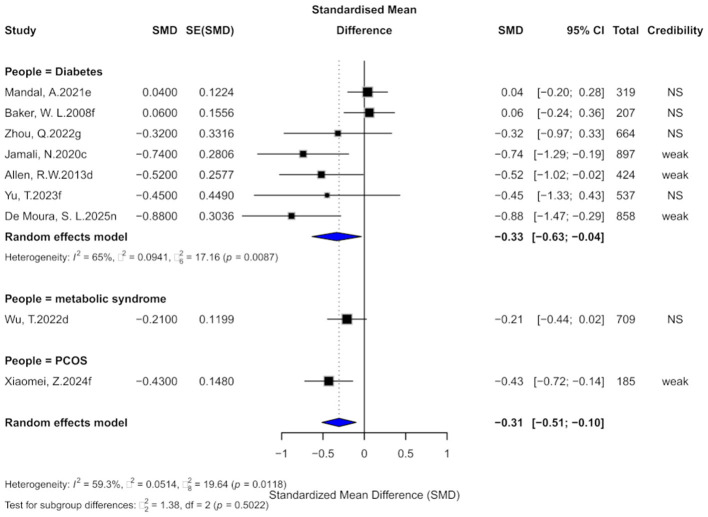
Forest plot of the effect of cinnamon supplementation on LDL in patients with metabolic diseases.

Nine comparisons assessed the effect of cinnamon supplementation on LDL levels. The pooled analysis indicated a statistically significant reduction (SMD = −0.31, 95% CI: −0.51 to −0.10), with moderate heterogeneity (*I*^2^ = 59.3%; Figure 9). Among the included studies, 66.7% exhibited substantial within-study heterogeneity (*I*^2^ > 50%). One study showed evidence of small-study effects, and three studies demonstrated excess significance bias. Egger's test did not indicate significant publication bias (bias = −2.34, *p* = 0.067), though the *p*-value was near the significance threshold. After applying the trim-and-fill method and imputing four potentially missing studies, the effect size became non-significant (SMD = −0.15, 95% CI: −0.41 to 0.12), with a slight increase in heterogeneity (*I*^2^ = 65.5%).

Regarding the level of evidence, 44.4% of comparisons were rated as “weak,” while the rest were “non-significant,” with none reaching the “suggestive” or higher level. All 95% prediction intervals included the null value. Statistically, 44.4% of comparisons were significant at *p* < 0.05, but none remained significant at the stricter threshold of *p* < 0.001.

Subgroup analyses revealed significant effects in patients with diabetes (SMD = −0.33) and PCOS (SMD = −0.43), whereas no meaningful effect was observed in those with metabolic syndrome. However, no significant subgroup differences were found (χ^2^ = 1.38, *p* = 0.5022).

### 3.8 Cinnamon and SBP outcomes

**Figure 10:**
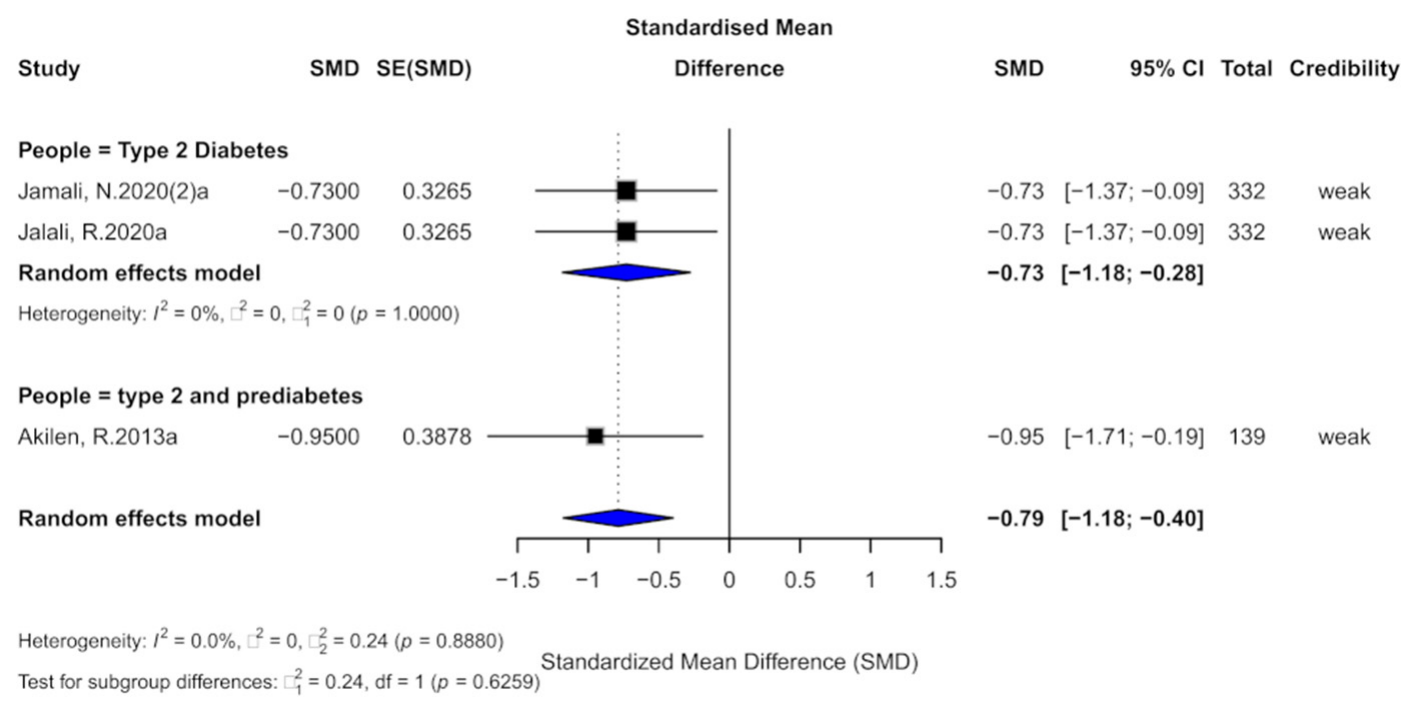
Forest plot of the effect of cinnamon supplementation on SBP in patients with metabolic diseases.

Three studies evaluated the effect of cinnamon supplementation on systolic blood pressure (SBP). The pooled analysis demonstrated a significant reduction in SBP (SMD = −0.79, 95% CI: −1.18 to −0.40), with very low overall heterogeneity (*I*^2^ = 0%; Figure 10). However, substantial within-study heterogeneity was observed across all included studies (*I*^2^ > 50%). No small-study effects were detected, but two studies exhibited evidence of excess significance bias. Due to the limited number of studies (*k* = 3), Egger's regression test could not be reliably performed. The trim-and-fill analysis did not suggest substantial funnel plot asymmetry, however, given the small sample size, the power to detect publication bias was limited, and the findings should be interpreted with caution.

In terms of the credibility of evidence, all three studies were classified as providing “weak” evidence. The 95% prediction intervals for all studies included the null value, and although all results were statistically significant at the *p* < 0.05 level, none reached the threshold of high significance (*p* < 0.001).

### 3.9 Cinnamon and DBP outcomes

**Figure 11:**
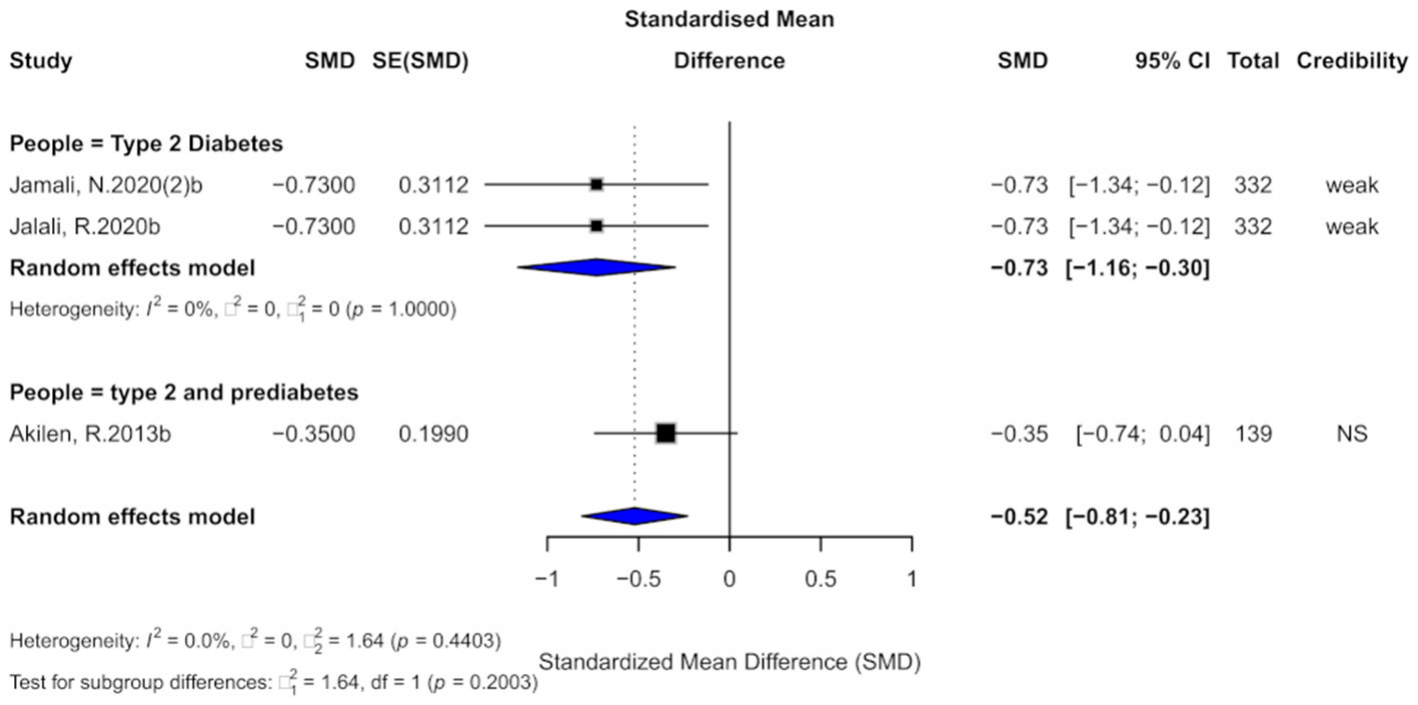
Forest plot of the effect of cinnamon supplementation on DBP in patients with metabolic diseases.

Three studies assessed the effect of cinnamon supplementation on DBP. The pooled analysis showed a significant reduction in DBP (SMD = −0.52, 95% CI: −0.81 to −0.23), with very low overall heterogeneity (*I*^2^ = 0%; Figure 11). However, two individual studies exhibited substantial within-study heterogeneity (*I*^2^ > 50%). No small-study effects were detected across the three studies, although one study demonstrated excess significance bias. The trim-and-fill analysis imputed two potentially missing studies, suggesting the possibility of publication bias. Due to the limited number of included studies (*k* = 3), Egger's regression test lacked sufficient power and was therefore not reliably performed. Overall, the current evidence is limited, and the findings should be interpreted with caution.

In terms of evidence credibility, two studies provided “weak” evidence, while one was classified as “non-significant.” The 95% prediction intervals for all studies included the null value. Although all three studies reported statistically significant results at *p* < 0.05, none reached the threshold of high significance (*p* < 0.001).

### 3.10 Cinnamon and BW outcomes

**Figure 12:**
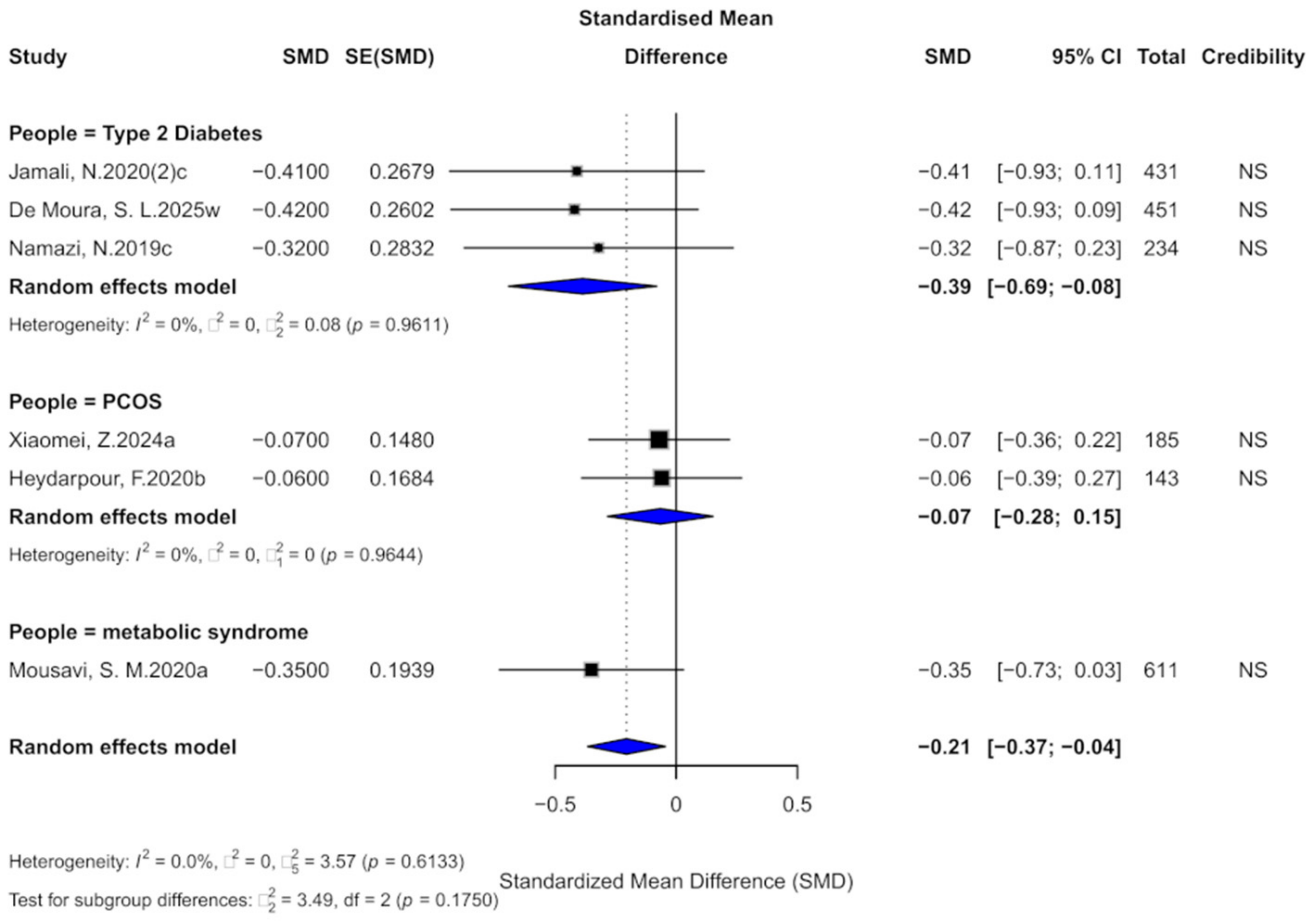
Forest plot of the effect of cinnamon supplementation on BW in patients with metabolic diseases.

Six comparisons evaluated the effect of cinnamon supplementation on BW, yielding a small but statistically significant effect (SMD = −0.21, 95% CI: −0.37 to −0.04), with low heterogeneity across studies (*I*^2^ = 0%; Figure 12). In terms of evidence grading, all comparisons were classified as “non-significant,” with none reaching the “weak” or higher level. Therefore, the current evidence does not support a significant effect of cinnamon supplementation on BW in patients with metabolic diseases.

### 3.11 Cinnamon and BMI outcomes

**Figure 13:**
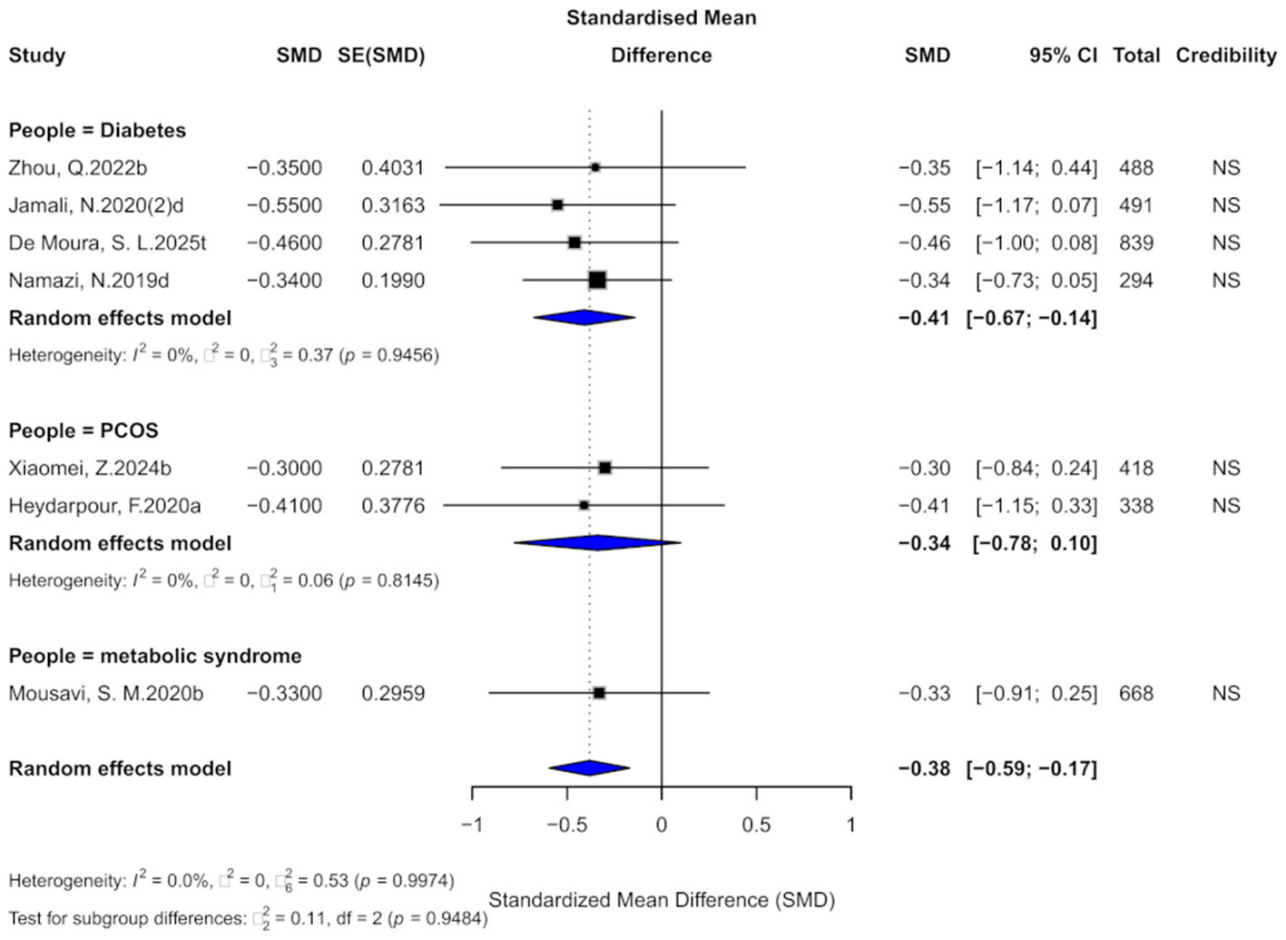
Forest plot of the effect of cinnamon supplementation on BMI in patients with metabolic diseases.

Seven comparisons assessed the impact of cinnamon supplementation on BMI, revealing a moderate and statistically significant reduction (SMD = −0.38, 95% CI: −0.59 to −0.17), with low heterogeneity observed across studies (*I*^2^ = 0%; Figure 13). Regarding evidence grading, all comparisons were categorized as “non-significant,” with none reaching the “weak” or higher level. Thus, current evidence does not provide strong support for a meaningful effect of cinnamon supplementation on BMI in individuals with metabolic diseases.

### 3.12 Cinnamon and adverse events outcomes

Among the 21 meta-analyses included in this study, only one reported on adverse events associated with cinnamon supplementation, with a relative risk of 0.83 (95% CI: 0.22–3.07). The level of evidence was classified as “non-significant,” and no definitive conclusions can be drawn at this time.

### 3.13 Re-estimation of effect sizes and credibility ceiling analysis results

Nr, Ns, and R were 209, 45, and 21, respectively, yielding a CCA value of 18.2%, which indicates a high degree of overlap among the included meta-analyses. Given the substantial redundancy, excluding overlapping reviews could have resulted in the omission of important studies and introduced selection bias. Therefore, we extracted and synthesized all relevant original studies from the included meta-analyses to conduct a reanalysis, aiming to provide a more comprehensive and less biased assessment of the current evidence.

The pooled effect estimates of cinnamon supplementation on various metabolic outcomes in patients with metabolic diseases based on the reanalysis are presented in Table 3. Subgroup analyses were also performed according to the dosage and duration of supplementation, with detailed results shown in Table 3.

**Table 3:** Results after reanalysis.

**Variable**	**Subgroup**	**SMD (95% CI)**	**Credibility**
FBG		−0.74 (−0.99, −0.48)	Highly suggestive
FBG	≤ 1.5 g/day	−0.60 (−0.91, −0.29)	Suggestive
FBG	>1.5 g/day	−1.02 (−1.45, −0.58)	Weak
FBG	≤ 2 months	−0.86 (−1.26, −0.46)	Suggestive
FBG	>2 months	−0.59 (−0.77, −0.40)	Highly suggestive
Hba1c		−0.09 (−0.98, 0.80)	NS
Hba1c	≤ 1.5 g/day	0.16 (−1.31, 1.64)	NS
Hba1c	>1.5 g/day	−0.32 (−0.62, −0.02)	Weak
Hba1c	≤ 2 months	1.29 (−1.65, 4.22)	NS
Hba1c	>2 months	−0.52 (−0.75, −0.29)	Suggestive
HOMA-IR		−1.42 (−2.97, 0.14)	NS
HOMA-IR	≤ 1.5 g/day	−1.69 (−3.64, 0.25)	NS
HOMA-IR	>1.5 g/day	−0.41 (−0.68, −0.14)	Weak
HOMA-IR	≤ 2 months	−0.42 (−0.61, −0.23)	Weak
HOMA-IR	>2 months	−2.65 (−5.95, 0.64)	NS
CHOL		−0.98 (−1.57, −0.39)	Suggestive
CHOL	≤ 1.5 g/day	−0.73 (−1.36, −0.10)	Weak
CHOL	>1.5 g/day	−1.58 (−2.91, −0.25)	Weak
CHOL	≤ 2 months	−1.31 (−2.26, −0.37)	Weak
CHOL	>2 months	−0.68 (−1.43, 0.07)	NS
TG		−0.56 (−0.90, −0.21)	Suggestive
TG	≤ 1.5 g/day	−0.43 (−0.83, −0.03)	Weak
TG	>1.5 g/day	−0.84 (−1.52, −0.07)	Weak
TG	≤ 2 months	−0.72 (−1.28, −0.16)	Weak
TG	>2 months	−0.43 (−0.87, 0.01)	NS
HDL		0.15 (−0.27, 0.57)	NS
HDL	≤ 1.5 g/day	0.20 (−0.38, 0.78)	NS
HDL	>1.5 g/day	−0.02 (−0.26, 0.22)	NS
HDL	≤ 2 months	0.13 (−0.19, 0.45)	NS
HDL	>2 months	0.20 (−0.49, 0.89)	NS
LDL		−0.59 (−0.98, −0.20)	Weak
LDL	≤ 1.5 g/day	−0.42 (−0.85, 0.01)	NS
LDL	>1.5 g/day	−1.03 (−1.92, −0.14)	Weak
LDL	≤ 2 months	−0.77 (−1.36, −0.18)	Weak
LDL	>2 months	−0.43 (−0.97, 0.11)	NS
SBP		−0.73 (−1.28, −0.18)	Weak
SBP	≤ 1.5 g/day	−0.87 (−2.34, 0.61)	NS
SBP	>1.5 g/day	−0.67 (−1.28, −0.05)	Weak
SBP	≤ 2 months	−0.95 (−1.68, −0.22)	Weak
SBP	>2 months	−0.52 (−1.40, 0.37)	NS
DBP		−0.65 (−1.21, −0.10)	Weak
DBP	≤ 1.5 g/day	−1.20 (−2.50, −0.10)	Weak
DBP	>1.5 g/day	−0.35 (−0.75, 0.04)	NS
DBP	≤ 2 months	−0.35 (−0.77, 0.07)	NS
DBP	>2 months	−0.95 (−1.96, 0.06)	NS
BW		−0.29 (−0.60, 0.01)	NS
BW	≤ 1.5 g/day	−0.28 (−0.80, 0.25)	NS
BW	>1.5 g/day	−0.31 (−0.72, 0.10)	NS
BW	≤ 2 months	−0.51 (−1.03, 0.01)	NS
BW	>2 months	−0.04 (−0.27, 0.19)	NS
BMI		−0.25 (−0.57, 0.06)	NS
BMI	≤ 1.5 g/day	−0.26 (−0.72, 0.19)	NS
BMI	>1.5 g/day	−0.21 (−0.53, 0.11)	NS
BMI	≤ 2 months	−0.42 (−1.03, 0.19)	NS
BMI	>2 months	−0.04 (−0.20, 0.12)	NS

NS, not-significant.

## 4 Discussion

This umbrella review highlights the potential role of cinnamon supplementation as a complementary approach for managing metabolic outcomes in patients with metabolic diseases. While variations in cinnamon form, dosage, intervention duration, and underlying disease conditions may contribute to heterogeneity across studies, the overall evidence suggests that cinnamon could improve glucose metabolism, lipid profiles, and other metabolic parameters. These findings underscore the promise of cinnamon as an adjunctive nutritional strategy, while also emphasizing the need for cautious interpretation.

In terms of glucose metabolism, this study selected FBG, HbA1c, HOMA-IR as the primary evaluation indicators. The results suggest that cinnamon supplementation may improve FBG in patients with metabolic diseases, with the highest level of evidence rated as “suggestive.” Given the substantial overlap among the original studies, we reanalyzed all relevant primary data, which continued to support the beneficial effect of cinnamon on glycemic control, with the evidence level upgraded to “highly suggestive.” Furthermore, higher doses (>1.5 g/day) and shorter intervention durations ( ≤ 2 months) were associated with more pronounced improvements, suggesting that short-term, high-dose interventions may yield more clinically meaningful benefits. Although cinnamon supplementation also showed trends toward improvement in HbA1c and HOMA-IR, the supporting evidence for these outcomes was consistently rated as “weak,” and reanalysis of the original data rendered the overall effects non-significant. Therefore, caution is warranted when interpreting the effects of cinnamon on HbA1c and HOMA-IR, and further high-quality studies are required to confirm these findings.

Several existing reviews and original studies have proposed the potential antidiabetic mechanisms of cinnamon (30–34). Purified cinnamon extract (CE) and cinnamon polyphenols (CP) have been shown to upregulate insulin receptor β (IRβ) and glucose transporter 4 (GLUT4) protein expression in 3T3-L1 adipocytes, thereby enhancing insulin signaling and glucose uptake. CP also increases GLUT4 levels, suggesting insulin-like activity and long-term regulation of glucose transport (33–36). Insulin resistance is associated with impaired GLUT4 translocation due to disrupted tyrosine phosphorylation of insulin receptor substrates (IRS) (35, 36). Methylhydroxychalcone polymer (MHCP), a bioactive compound in cinnamon, mimics insulin action by activating the IRS–PI3K pathway, promoting glucose uptake and glycogen synthesis, and inhibiting glycogen synthase kinase-3β (GSK-3β) (33, 35, 37). Moreover, cinnamon suppresses hepatic gluconeogenesis by downregulating phosphoenolpyruvate carboxykinase (PEPCK) and glucose-6-phosphatase, and activates AMP-activated protein kinase (AMPK), leading to improved energy metabolism and upregulation of PPAR-α and PPAR-γ, which help modulate lipid and glucose metabolism (38–40). Additionally, cinnamon may exert glycemic benefits via gastrointestinal mechanisms, such as delaying gastric emptying and glucose absorption, and enhancing cellular glucose utilization (34, 41).

In terms of lipid metabolism, the findings suggest that cinnamon supplementation may lead to modest to moderate improvements in CHOL, TG, and LDL, although the highest level of evidence supporting these effects was rated as “weak.” No significant impact was observed on HDL. Subgroup analyses based on disease type indicated no substantial differences in the effects of cinnamon among patients with diabetes, metabolic syndrome, or PCOS. Following a re-analysis of all original study data, the beneficial effects on CHOL, TG, and LDL were further supported, and the evidence level for some outcomes was upgraded to “suggestive.” Additionally, subgroup analyses by dose and intervention duration showed that higher doses (>1.5 g/day) and shorter intervention periods ( ≤ 2 months) were associated with greater improvements in CHOL and TG.

Studies have shown that the lipid-lowering effects of cinnamon are mediated through multiple mechanisms. Firstly, cinnamon inhibits hepatic HMG-CoA reductase activity, thereby reducing endogenous cholesterol synthesis (42). It also promotes lipolysis, potentially by improving insulin resistance and suppressing the overproduction of intestinal apoB48-containing lipoproteins, thus contributing to lipid metabolism regulation (42, 43). Moreover, cinnamon is rich in polyphenolic compounds, which not only inhibit intestinal cholesterol absorption (44), but also upregulate the expression of peroxisome proliferator-activated receptor alpha (PPAR-α) in adipose tissue. This leads to enhanced lipoprotein lipase activity and improved uptake and metabolism of free fatty acids (32, 45, 46). Cinnamon also facilitates lipid metabolism via activation of antioxidant pathways. Animal studies have demonstrated that cinnamon supplementation significantly increases the expression of nuclear factor erythroid 2–related factor 2 (Nrf2) and its downstream effector heme oxygenase-1 (HO-1) (47). In addition, *in vitro* research indicates that cinnamic acid can inhibit pancreatic lipase activity, thereby reducing the hydrolysis of dietary TG and subsequent intestinal absorption of fatty acids, ultimately contributing to decreased LDL-C and increased HDL-C levels (48, 49). S-(+)-Linalool, a major component of cinnamon, has also been shown to significantly reduce plasma triglyceride (TG) levels and exert anti-adipogenic effects by inhibiting lipid accumulation in 3T3-L1 adipocytes (50).

The meta-analysis in this umbrella review found no statistically significant effects of cinnamon supplementation on BMI and BW, with all seven included outcomes being non-significant. Similarly, after re-extracting and reanalyzing data from the original studies, no significant differences were observed. This finding is consistent with the study by Namazi et al. (51) which also reported no significant improvements in BW or BMI following cinnamon supplementation. However, it contrasts with the meta-analysis by Mousavi et al. (52) which included 12 RCTs and found that cinnamon significantly reduced BW, BMI, waist circumference, and body fat percentage—particularly among individuals aged < 50 years or those with a baseline BMI ≥30 kg/m^2^. In addition, the umbrella review by Keramati et al. (53) supported the beneficial effects of cinnamon in significantly reducing BW and BMI. These discrepancies may be attributed to methodological differences. Unlike previous studies that commonly used mean difference (MD) or weighted mean difference (WMD) as effect sizes, the present study applied SMD for data synthesis. Moreover, we re-extracted baseline and post-intervention values from the original studies and calculated effect sizes based on pre- and post-intervention changes, rather than using only the final endpoint values. Such methodological distinctions may partly explain the inconsistent results across studies.

In terms of blood pressure regulation, the findings of this study indicate that cinnamon supplementation exerts a moderate to strong lowering effect on both SBP and DBP. However, the quality of evidence was mostly rated as “weak.” After re-extracting and re-analyzing all original study data, the SMDs remained largely unchanged, and the strength of evidence was consistent, suggesting that the conclusions are relatively robust. Subgroup analyses further revealed that the significant reduction in SBP was primarily observed in studies using a daily dose >1.5 g and an intervention duration of no more than 2 months. In contrast, the reduction in DBP was more pronounced in studies using a lower daily dose ( ≤ 1.5 g). This dose-response relationship suggests that cinnamon's effects on blood pressure may involve different mechanisms or threshold effects, warranting further investigation.

Oxidative stress plays a critical role in the onset and progression of diabetes and cardiovascular diseases (54). Evidence suggests that cinnamon can enhance the antioxidant status in individuals with metabolic syndrome, attenuate free radical generation (55), and lower plasma malondialdehyde concentrations (56), thereby reducing lipid peroxidation and potentially contributing to blood pressure regulation. With respect to vascular function, cinnamon has been shown to increase serum nitric oxide (NO) levels (57) and promote its production (58), facilitating vasodilation, while also stimulating the release of calcitonin gene-related peptide (CGRP) (59) and improving arterial wall compliance (60). It can suppress vascular smooth muscle cell proliferation (61) and downregulate the transcription and mRNA expression of endothelial factors, leading to reduced expression of vascular cell adhesion molecule-1 (VCAM-1) and SICAM-1 (62). On the metabolic side, cinnamon improves insulin resistance (55), helps maintain normal vascular contractility through modulation of Ca^2+^ influx (24), and alleviates hyperuricemia (63). Additionally, it may reduce sympathetic nerve activity (64) and mitigate resting tachycardia, neural hyperexcitability, and elevated plasma norepinephrine (64). Collectively, these mechanisms may act synergistically to lower blood pressure, with effects potentially more pronounced in individuals with diabetes or metabolic syndrome.

Due to the limited number of meta-analyses reporting adverse effects of cinnamon, with only one relevant meta-analysis included in this study (65), data re-pooling was not feasible. In the included meta-analysis, two primary studies reported adverse events in participants receiving cinnamon at a dose of 1 g: one case of rash (66) and one case of hives (67). Two other primary studies reported adverse events in the control groups: one case of nausea (68) and one case of mild gastric pain lasting 2 days (69). Overall, adverse events associated with oral cinnamon were infrequent and generally mild. The U.S. Food and Drug Administration (FDA) has classified cinnamon as Generally Recognized As Safe (GRAS). Current evidence indicates that cinnamon is well-tolerated at daily doses up to 6 grams, while higher doses may cause mild and self-limiting gastrointestinal or skin reactions (7, 70, 71). Systematic reviews also support its safety as a dietary component or herbal supplement (7, 65). Human safety data are limited, with most evidence derived from *in vitro* and animal studies, which suggest that high coumarin content may lead to hepatotoxicity, bleeding risks, allergic reactions, and potential carcinogenicity (72, 73). Overall, cinnamon is considered safe at appropriate doses, but its long-term safety requires further clinical investigation.

Recently, Qin et al. (74) confirmed in a review that cinnamon and its active components exert beneficial effects on multiple parameters related to metabolic syndrome, including insulin sensitivity, blood glucose levels, lipid regulation, antioxidant capacity, inflammation, blood pressure, and weight management, which aligns broadly with the findings of our study. Furthermore, the combined use of cinnamon with a low-carbohydrate ketogenic diet (LCKD) has shown potential in improving glycemic and blood pressure control (75). As a low-cost and readily accessible natural product, cinnamon demonstrates promising clinical application prospects and may serve as a complementary therapy and nutritional intervention. Future well-designed, high-quality clinical trials are warranted to further validate its long-term efficacy, safety, and underlying mechanisms, thereby facilitating its broader application in the management of metabolic diseases.

This umbrella review has several strengths. First, it comprehensively synthesizes published meta-analyses examining the association between cinnamon supplementation and metabolic outcomes in patients with metabolic diseases, covering a wide range of indicators. Second, a rigorous and systematic search strategy was employed across multiple databases, with study selection and data extraction independently conducted by two researchers, ensuring quality and objectivity. Third, pooled effect sizes for each meta-analysis were recalculated using a random-effects model, alongside assessments of heterogeneity, small-study effects, and excess significance bias, thereby enhancing the reliability of the findings. Fourth, to address overlap among included studies, we reanalyzed all original study data to minimize bias from duplicated data inclusion. Lastly, this review used pre- and post-intervention changes as the basis for data synthesis rather than relying solely on post-intervention values, which better controls for baseline differences.

Nevertheless, several limitations exist. First, due to methodological constraints, only meta-analyses with complete individual study data were included, potentially excluding relevant studies lacking comprehensive data. Second, despite stringent inclusion criteria, residual bias cannot be entirely ruled out, given heterogeneity in patient baseline characteristics, cinnamon varieties, and preparation methods. Finally, some meta-analyses included fewer than 10 studies, which limits the statistical power to detect small-study effects and excess significance bias, complicating the identification of potential sources of bias.

## 5 Conclusion

Cinnamon supplementation, as a natural metabolic modulator, has been extensively studied for its effects on metabolic disorder-related parameters. This study comprehensively evaluated the associations between cinnamon supplementation and metabolic indicators—including blood glucose, lipid profiles, blood pressure, and body weight—in patients with metabolic syndrome. The results demonstrated that cinnamon supplementation significantly improved fasting blood glucose and lipid levels, particularly among individuals with diabetes and metabolic syndrome. Subgroup analyses indicated that higher doses (>1.5 g/day) and shorter intervention durations ( ≤ 2 months) were more likely to yield clinically meaningful improvements. Additionally, cinnamon showed potential benefits in modulating insulin resistance, oxidative stress, and blood pressure regulation. These findings underscore the promising role of cinnamon as an adjunctive therapy and nutritional intervention in managing metabolic diseases.

## Data Availability

The original contributions presented in the study are included in the article/supplementary material, further inquiries can be directed to the corresponding authors.
